# STAT3 promotes a youthful epigenetic state in articular chondrocytes

**DOI:** 10.1111/acel.13773

**Published:** 2023-01-13

**Authors:** Arijita Sarkar, Nancy Q. Liu, Jenny Magallanes, Jade Tassey, Siyoung Lee, Ruzanna Shkhyan, Youngjoo Lee, Jinxiu Lu, Yuxin Ouyang, Hanhan Tang, Fangzhou Bian, Litao Tao, Neil Segil, Jason Ernst, Karen Lyons, Steve Horvath, Denis Evseenko

**Affiliations:** ^1^ Department of Orthopaedic Surgery, Keck School of Medicine of USC University of Southern California (USC) Los Angeles California USA; ^2^ Department of Biomedical Sciences Creighton University Nebraska Omaha USA; ^3^ Department of Stem Cell and Regenerative Medicine University of Southern California Los Angeles California USA; ^4^ Eli and Edythe Broad Center University of Southern California Los Angeles California USA; ^5^ Department of Biological Chemistry University of California Los Angeles California USA; ^6^ Eli and Edythe Broad Center of Regenerative Medicine and Stem Cell Research at UCLA Los Angeles California USA; ^7^ Computer Science Department University of California Los Angeles California USA; ^8^ Jonsson Comprehensive Cancer Center, University of California Los Angeles California USA; ^9^ Molecular Biology Institute, University of California Los Angeles California USA; ^10^ Department of Computational Medicine University of California Los Angeles California USA; ^11^ Department of Orthopaedic Surgery University of California Los Angeles California USA; ^12^ Department of Biostatistics, Fielding School of Public Health University of California Los Angeles California USA; ^13^ Department of Human Genetics, David Geffen School of Medicine University of California Los Angeles California USA

**Keywords:** chondrocyte aging, DNA methylation, epigenetics, osteoarthritis, regeneration, STAT3

## Abstract

Epigenetic mechanisms guiding articular cartilage regeneration and age‐related disease such as osteoarthritis (OA) are poorly understood. STAT3 is a critical age‐patterned transcription factor highly active in fetal and OA chondrocytes, but the context‐specific role of STAT3 in regulating the epigenome of cartilage cells remain elusive. In this study, DNA methylation profiling was performed across human chondrocyte ontogeny to build an epigenetic clock and establish an association between CpG methylation and human chondrocyte age. Exposure of adult chondrocytes to a small molecule STAT3 agonist decreased DNA methylation, while genetic ablation of STAT3 in fetal chondrocytes induced global hypermethylation. CUT&RUN assay and subsequent transcriptional validation revealed DNA methyltransferase 3 beta (DNMT3B) as one of the putative STAT3 targets in chondrocyte development and OA. Functional assessment of human OA chondrocytes showed the acquisition of progenitor‐like immature phenotype by a significant subset of cells. Finally, conditional deletion of *Stat3* in cartilage cells increased DNMT3B expression in articular chondrocytes in the knee joint *in vivo* and resulted in a more prominent OA progression in a post‐traumatic OA (PTOA) mouse model induced by destabilization of the medial meniscus (DMM). Taken together these data reveal a novel role for STAT3 in regulating DNA methylation in cartilage development and disease. Our findings also suggest that elevated levels of active STAT3 in OA chondrocytes may indicate an intrinsic attempt of the tissue to regenerate by promoting a progenitor‐like phenotype. However, it is likely that chronic activation of this pathway, induced by IL‐6 cytokines, is detrimental and leads to tissue degeneration.

## Graphical abstract

This study uncovers a context‐specific role of STAT3 in regulating chondrocyte development and disease suggesting that activation of STAT3 signaling may reduce epigenetic age by decreasing DNA methylation in adult cells and may be part of the regenerative mechanism induced by inflammatory processes. The novel DNA methylation‐based epigenetic clock developed will help researchers to capture pivotal aspects of biological age in adult chondrocytes. Finally, conditional deletion of *Stat3* in cartilage cells resulted in a more prominent OA progression in a post‐traumatic osteoarthritic mouse model.

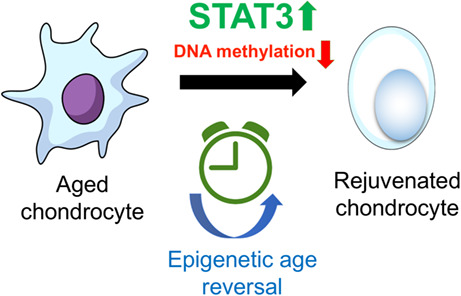

## 1 INTRODUCTION

Articular chondrocytes have limited potential for intrinsic healing and repair (Sophia Fox et al., 2009). Loss and degradation of articular chondrocytes is a significant cause of musculoskeletal morbidity (Sophia Fox et al., 2009). With aging, the regenerative potential of chondrocytes decreases, accompanied by significant changes in extracellular matrix composition, leading to inferior mechanical and structural properties, and surface fibrillation (Martin & Buckwalter, 2002). During osteoarthritis (OA) progression, activated chondrocytes expressing some immature cell markers have been reported (Sandell & Aigner, 2001). Unfortunately, this regenerative process, which results in the activation of an inflammatory response, is oftentimes unsuccessful, resulting in unfavorable outcomes, such as fibrosis or tissue degeneration. Although the cellular and molecular mechanisms for chondrocyte regeneration are poorly understood, it is presumed to involve many molecular pathways (Martel‐Pelletier et al., 2008). For example, degenerative diseases associated with aging, such as OA, are known to result from several factors including epigenetic changes (Fathollahi et al., 2019).

STAT3 is a well‐known master transcriptional factor that participates in a repertoire of signaling pathways in various tissues and contexts (Zhang et al., 2019). STAT3 signaling mediates numerous biological functions; its pro‐inflammatory role has been widely explored in various diseases including OA (Wang et al., 2020). However, STAT3 pathways are not only involved in pathology, inflammation and malignant transformation, but are also implicated in regulating stemness, development and regeneration of tissues and organs (Nakao et al., 2020). STAT3 also regulates chromatin accessibility via DNA methylation (Zhang et al., 2005). DNA methylation is a crucial player in the epigenetic regulation of aging (Gentilini et al., 2013). Because DNA methylation changes are reversible, they are an attractive therapeutic target for age‐related diseases, including OA. The dynamics of methylation in aging have impelled researchers to develop ‘epigenetic clocks’ as the new standard to accurately predict biological age (Horvath, 2013). However, the impact of DNA methylation on chondrocyte development across human ontogeny has not been studied to date.

Although STAT3 is well‐known as a key driver of inflammation in disease of the synovial joints, our recent studies have shown that the levels of active phosphorylated STAT3 (pSTAT3) are higher in developing fetal chondrocytes as compared to adult (Shkhyan et al., 2018). However, the transcriptional targets of STAT3 in human chondrocytes at different developmental stages and their potential roles in fetal chondrocytes have not been explored. In addition, it is still unknown whether STAT3's transcriptional targets are identical to those in early development and chronic inflammatory diseases, including OA. Finally, the impact of STAT3 on the epigenome in articular chondrocytes is unknown.

Here, we study the dynamic genome‐wide DNA methylation profile of human chondrocytes across ontogeny and identify fundamental factors involved in regulating chondrocyte aging in humans. We have determined a correlation between methylation of specific CpG sites and chondrocyte age. In addition, we investigate the enrichment of chromatin states in these age‐correlated CpGs. A novel epigenetic clock was built for adult human chondrocytes that accurately predicts epigenetic age. We utilized this clock to gain further insight into the effect of a small molecule STAT3 agonist, which decreased epigenetic age of aged adult chondrocytes. Moreover, the impact of genetic manipulation of STAT3 on genome‐wide DNA methylation was determined. Thereafter, we explored the putative binding targets of STAT3 in developing human chondrocytes, normal (healthy) articular chondrocytes and osteoarthritic (OA) chondrocytes. STAT3 modulation was associated with changes in the expression level of the *de novo* DNA methyltransferase, DNMT3B, in a context‐specific manner suggestive of its transcriptional regulation by STAT3. Furthermore, our data suggest that activating STAT3 signaling in OA chondrocytes might be responsible for inducing a proliferative capacity for tissue regeneration. In essence, these findings will serve as a foundation for understanding the regulatory mechanisms implicated in regulation of chondrocyte aging and help develop new therapeutic interventions to reverse or slow down age‐associated diseases, such as OA.

## 2 RESULTS

### 2.1 Epigenome‐wide association study (EWAS) identifies age‐correlated CpGs in non‐cultured human fetal and adult chondrocytes

**FIGURE 1:**
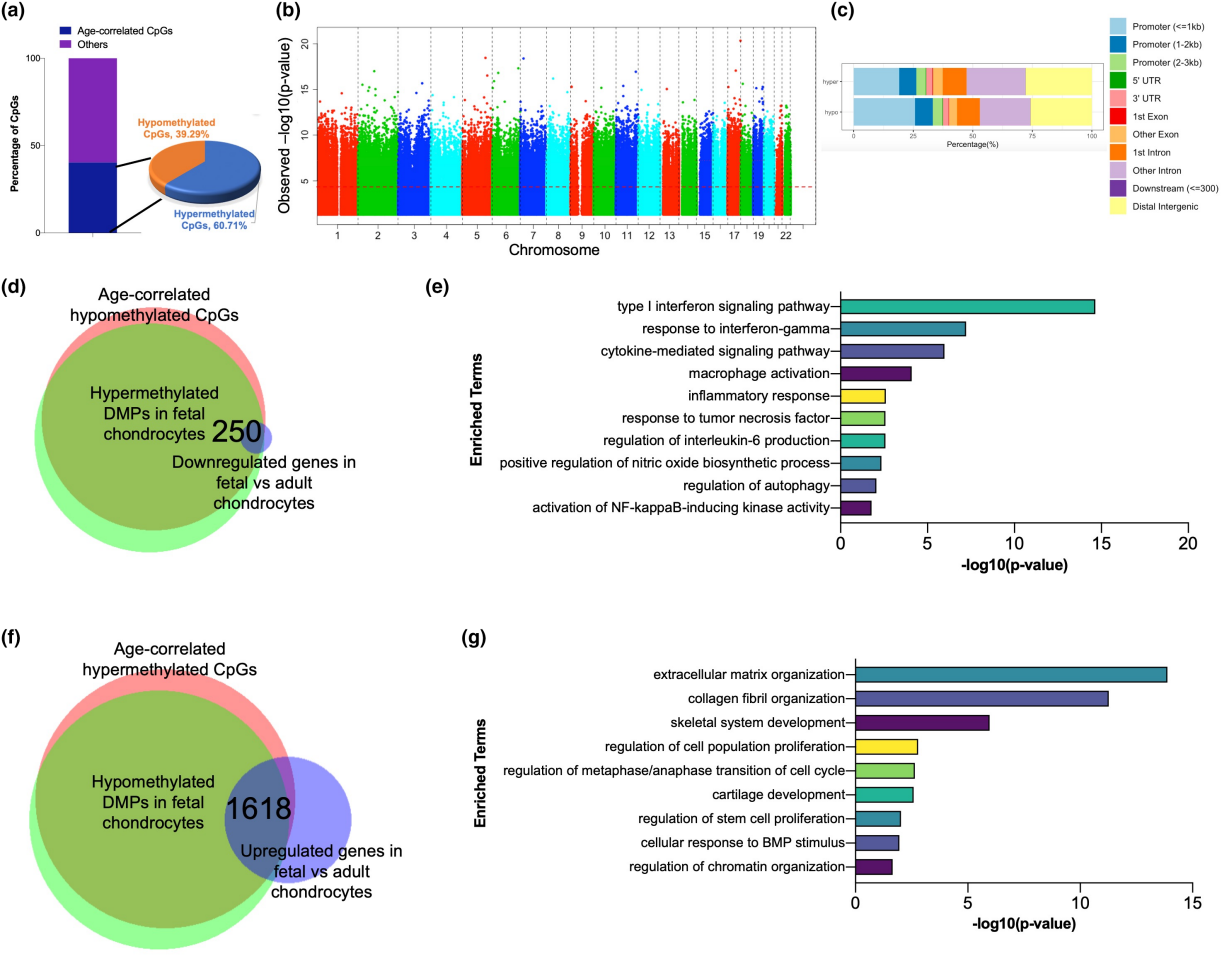
Epigenome‐wide association study for non‐cultured fetal and adult chondrocytes. (a) Bar diagram showing the percentage of age‐correlated CpGs among all significant CpGs. (b) Manhattan plot showing chromosomal locations of age‐correlated CpGs along with −log_10_(*p* values) for association at each locus. The red dotted line indicates the p‐value threshold of 0.05. (c) Distribution of genomic features among age‐correlated CpGs. hyper = hypermethylated CpGs, hypo = hypomethylated CpGs. (d) Venn diagram showing the overlap between age‐correlated hypomethylated CpGs, differentially hypermethylated CpGs in fetal chondrocytes and differentially expressed genes in fetal chondrocytes when compared to adult chondrocytes. (e) Gene ontology enrichment analysis for the 250 genes obtained from (d). (f) Venn diagram showing the overlap between age‐correlated hypermethylated CpGs, differentially hypomethylated CpGs in fetal chondrocytes and differentially expressed genes in fetal chondrocytes when compared to adult chondrocytes. (g) Gene ontology enrichment analysis for the 1618 genes obtained from (f). DMPs, differentially methylated CpG probes

We performed DNA methylation profiling of non‐cultured human fetal (*n* = 8) and adult chondrocytes (*n* = 22) to identify regulatory genes associated with ontogeny specification (Figure S1). Evaluation of global methylation patterns (hypomethylation and hypermethylation) across the ontogeny revealed a correlation with chondrocyte age (Figure 1a, Table S1). The site‐specific genome‐wide pattern of DNA methylation showed a predominant proportion of age‐correlated CpG sites to be statistically significant (*p* < 0.05) (Figure 1b). These CpGs showing either gain or loss of methylation with age (i.e., hypermethylated or hypomethylated, respectively) were unevenly distributed across the genome (Figure 1c). Thereafter, we determined the differentially methylated CpGs in fetal chondrocytes when compared to adult chondrocytes. We investigated the overlap of genes associated with differentially methylated CpGs and age‐correlated CpGs in addition to differentially expressed genes in fetal vs adult chondrocytes. We identified 250 genes that lost methylation with age and are downregulated in fetal chondrocytes compared to adult chondrocytes (Figure 1d). Interestingly, these genes show enrichment of functions associated with inflammatory responses, NF–kB‐inducing kinase activity, macrophage activation, and interferon signaling pathway, indicating that progression of age is characterized by an increase in destructive responses (Figure 1e). Similarly, we determined that 1618 genes gained methylation with age and are upregulated in fetal chondrocytes when compared to adult chondrocytes (Figure 1f). Most of these genes were associated with skeletal system development, collagen fibril organization, extracellular matrix organization, and cell proliferation including chromatin organization, indicating regulation of homeostasis and anabolism in developing chondrocytes (Figure 1g). Overall, age‐correlated CpGs show a distinct methylation profile, which governs the ontogeny‐specific phenomenon of development.

### 2.2 Age‐correlated CpGs are associated with distinct chromatin signatures

It has been previously reported that DNA methylation patterning is governed by various chromatin states, such as histone modifications and nucleosome positioning (Robertson, 2002). In addition, various chromatin remodeling factors might interact with DNA methyltransferases, guide them to specific DNA sequences, and modulate transcriptional activation/repression. As mentioned previously, age‐correlated CpGs are associated with chromatin organization. Thus, we hypothesized that age‐correlated CpGs might be associated with distinct chromatin states in chondrocytes. Accordingly, we determined the chromatin states associated with age‐correlated CpGs (i.e., both hypermethylated (204,549 CpGs) and hypomethylated (132,383 CpGs)) in fetal and adult chondrocytes. To do this, we used the ChromHMM chromatin state model previously generated by our group (Ferguson et al., 2018) based on data from four histone modifications (H3K4me3, H3K27me3, H3K4me1, and H3K27ac) (Figure S2). We observed that CpGs in fetal chondrocytes, which gain methylation with age, show stronger enrichment for a poised promoter or bivalent state, characterized by the co‐existence of both activating (H3K4me3) and repressing (H3K27me3) marks. Interestingly, bivalent chromatin states have previously been known to be enriched in developmentally essential genes (Bernstein et al., 2006). CpGs in adult chondrocytes, which lose methylation with age, are most enriched for the active enhancer chromatin state, suggestive of transcriptional regulation from these regions. Of note, gain or loss of methylation in CpGs correlated with age in both fetal and adult chondrocytes show enrichment for chromatin states associated with enhancers (marked by H3K27ac) which likely reflects the fact that cell type specific enhancers are activated upon chondrocyte differentiation (Cheung et al., 2020). Together, these findings affirm that age‐correlated CpGs are intrinsically tied to distinct chromatin states.

### 2.3 A novel epigenetic clock for adult chondrocytes can accurately predict STAT3 agonist‐induced global hypomethylation

**FIGURE 2:**
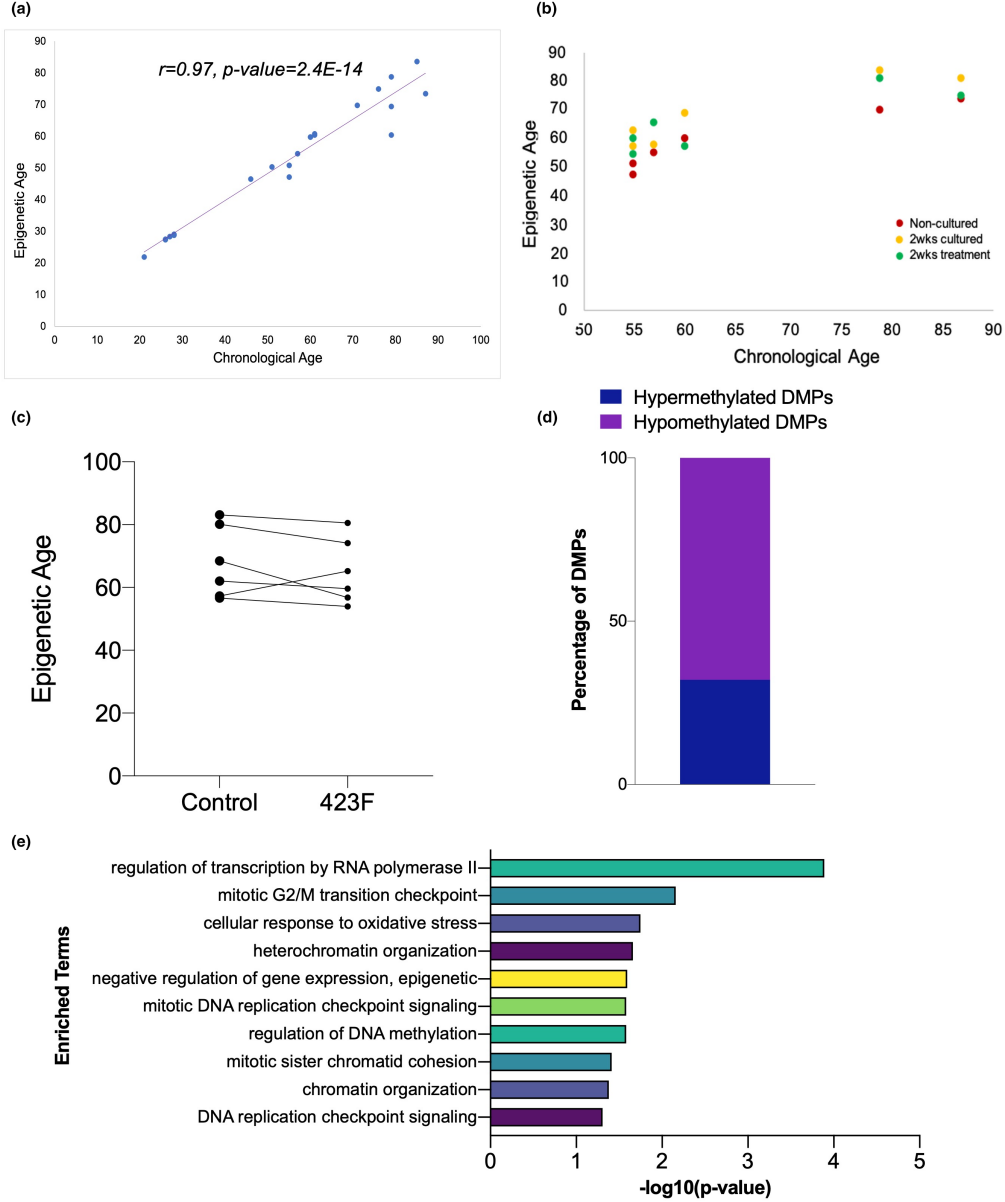
A novel epigenetic clock for adult chondrocytes. (a) Epigenetic clock for adult chondrocytes shows high correlation between epigenetic age and chronological age. (b,c) Administration of a small molecule STAT3 agonist to adult chondrocytes for 2 weeks lowers epigenetic age. (d) Differentially methylated CpGs in 2 weeks cultured treated chondrocytes compared to control samples show global gain in hypomethylation (e) Gene ontology enrichment analysis for the genes‐associated with differentially hypomethylated CpGs. DMPs, differentially methylated CpG probes

Since the late 1960s, a vast majority of literature describes DNA methylation levels as having strong effects on the aging of tissues and cells. DNA methylation based epigenetic clocks are the best biological age predictors to date (Jylhava et al., 2017). To the best of our knowledge, we, for the first time, have developed a novel epigenetic clock specific to human adult chondrocytes. This clock utilizes DNA methylation data to estimate the biological age of human adult chondrocytes with high accuracy (*r* = 0.97, *p*‐value = 2.4E‐14) (Figure 2a). Furthermore, we used this novel clock to accurately predict the epigenetic age of adult chondrocytes upon treatment with a STAT3 agonist.

STAT3 has been previously reported to be one of the key factors orchestrating the epigenetic regulatory networks in various normal and malignant stem and progenitor cells (Galoczova et al., 2018; Huynh et al., 2019; Tang et al., 2012; Yang et al., 2010). We assessed whether activation of STAT3 would affect the global DNA methylation profile in aged adult chondrocytes. Healthy adult articular chondrocytes have very low basal levels of STAT3 signaling. Hence, we treated aged adult chondrocytes (*n* = 6) with or without the STAT3 agonist, 423F (Flores et al., 2017; Shkhyan et al., 2018) for 2 weeks and performed DNA methylation profiling (Figure S3). Interestingly, based on the novel clock, 5 out of 6 tested adult chondrocyte donors showed a clear decrease in DNA methylation, i.e., a decrease in biological age upon treatment for 2 weeks (Figure 2b,c). To substantiate our results, we determined the differentially methylated CpGs between treated and control samples that were cultured for 2 weeks and observed a global hypomethylation (Figure 2d). In addition, these differentially hypomethylated CpGs are associated with genes involved in cell cycle, DNA methylation, and chromatin organization (Figure 2e). Taken together, these results suggest that pharmacological activation of STAT3 signaling in aged adult chondrocytes reduces their epigenetic age. These proof‐of‐concept studies open a new perspective for development of therapeutically relevant reprogramming agents for aged articular chondrocytes.

### 2.4 Genetic manipulation of STAT3 induces global hypermethylation in fetal chondrocytes

**FIGURE 3:**
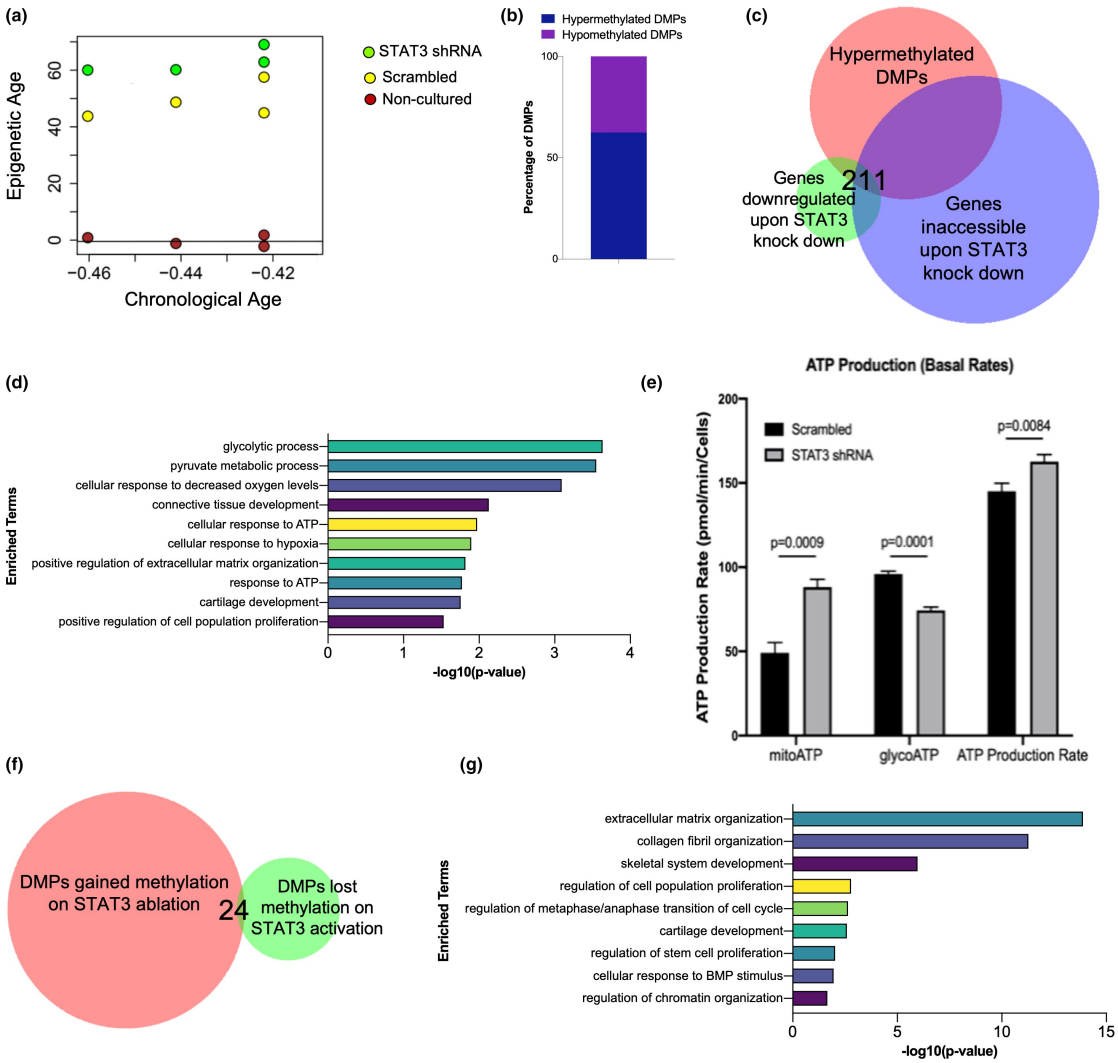
STAT3 knockdown induces genomic hypermethylation in fetal chondrocytes. (a) Fetal chondrocytes treated with STAT3 shRNA for 4 weeks increases epigenetic age. Chronological age of fetal chondrocytes are in negative as all age estimates are in units of years. (b) Bar diagram shows gain in hypermethylation in differentially methylated CpGs. (c) Venn diagram showing the overlap between genes associated with differentially methylated CpGs that gain methylation and the genes downregulated (i.e., genes with inaccessible chromatin) in STAT3 knocked down fetal chondrocytes. (d) Gene ontology enrichment analysis for genes obtained from (c). (e) Bioenergetic analysis using seahorse assay for STAT3 knocked down fetal chondrocytes. (f) Venn diagram showing overlap between CpGs which gain methylation in STAT3 ablated fetal chondrocytes and CpGs which lose methylation upon STAT3 activation in adult chondrocytes. (g) Gene ontology enrichment analysis for genes obtained from (f). DMPs, differentially methylated CpG probes

Based on the preceding pharmacological results, we hypothesized that genetic manipulation of STAT3 in fetal chondrocytes might also have an impact on genome‐wide DNA methylation. As shown by our group previously, fetal chondrocytes have very high basal levels of active STAT3 signaling (Shkhyan et al., 2018). We therefore transduced fetal chondrocytes with STAT3 shRNA (*n* = 4) and scrambled control (*n* = 4) (Figure S4) and performed DNA methylation profiling (Figure S5). Interestingly, STAT3 inhibition led to an increase in DNA methylation (i.e., increase in biological age) (Figure 3a). Next, we found that differentially methylated CpGs gained methylation (hypermethylated) in STAT3 knocked down fetal chondrocytes (Figure 3b). Furthermore, we explored the concordance between genes associated with differentially methylated CpGs that gain methylation and genes downregulated (i.e., genes with inaccessible chromatin) in STAT3 knocked down fetal chondrocytes (Liu, Lin, Li, Lu, Geng, Zhang, & Evseenko, 2022) (Figure 3c). The 211 genes obtained from this overlap revealed an association with connective tissue development, extracellular matrix organization, response to oxygen levels, response to ATP, and glycolytic processes (Figure 3d). Surprisingly, upon measuring the cellular bioenergetics of STAT3 inhibited fetal chondrocytes, we indeed observe a metabolic switch in basal ATP production rates consistent with a more differentiated phenotype during STAT3 inhibition (Figure 3e) as previously observed in other cell types (Flores et al., 2017). In summary, it can be concluded that upon STAT3 inhibition in fetal chondrocytes, there is a global gain in methylation that might contribute to epigenetic aging of these cells.

We predict STAT3 to be crucial for maintaining global methylation patterns, and next determined whether gain in methylation in STAT3‐ablated fetal chondrocytes happens at the same CpGs that lose methylation upon STAT3 activation in adult chondrocytes (Figure 3f). We determined the genes associated with these CpGs and performed functional enrichment analysis. The results indicated that gain or loss of methylation occurs mainly in genes associated with critical processes during aging, including cartilage development, skeletal system development, extracellular matrix organization, cell proliferation, cell cycle and chromatin organization (Figure 3g).

### 2.5 Assessing genome‐wide putative STAT3 targets in human developing chondrocytes, healthy adult articular chondrocytes and osteoarthritic chondrocytes

STAT3 exhibits a plethora of context‐specific functions in skeletal development, inflammation, and neoplastic growth (Sims, 2020). It also regulates the methylation of CpG sites by interacting with DNA methyltransferases (Zhang et al., 2005). As mentioned previously, our group has observed STAT3 to be highly expressed in fetal chondrocytes compared to healthy adult cells (Shkhyan et al., 2018). In addition, pSTAT3 is highly expressed in OA chondrocytes in comparison to healthy adult cells (Figure S6). Hence, it is plausible that although STAT3 is highly expressed in fetal and OA chondrocytes when compared to healthy adult cells, the downstream outcomes of STAT3 are different in each context. This led us to hypothesize that STAT3 has different context‐specific transcriptional targets that differ in development and disease.

**FIGURE 4:**
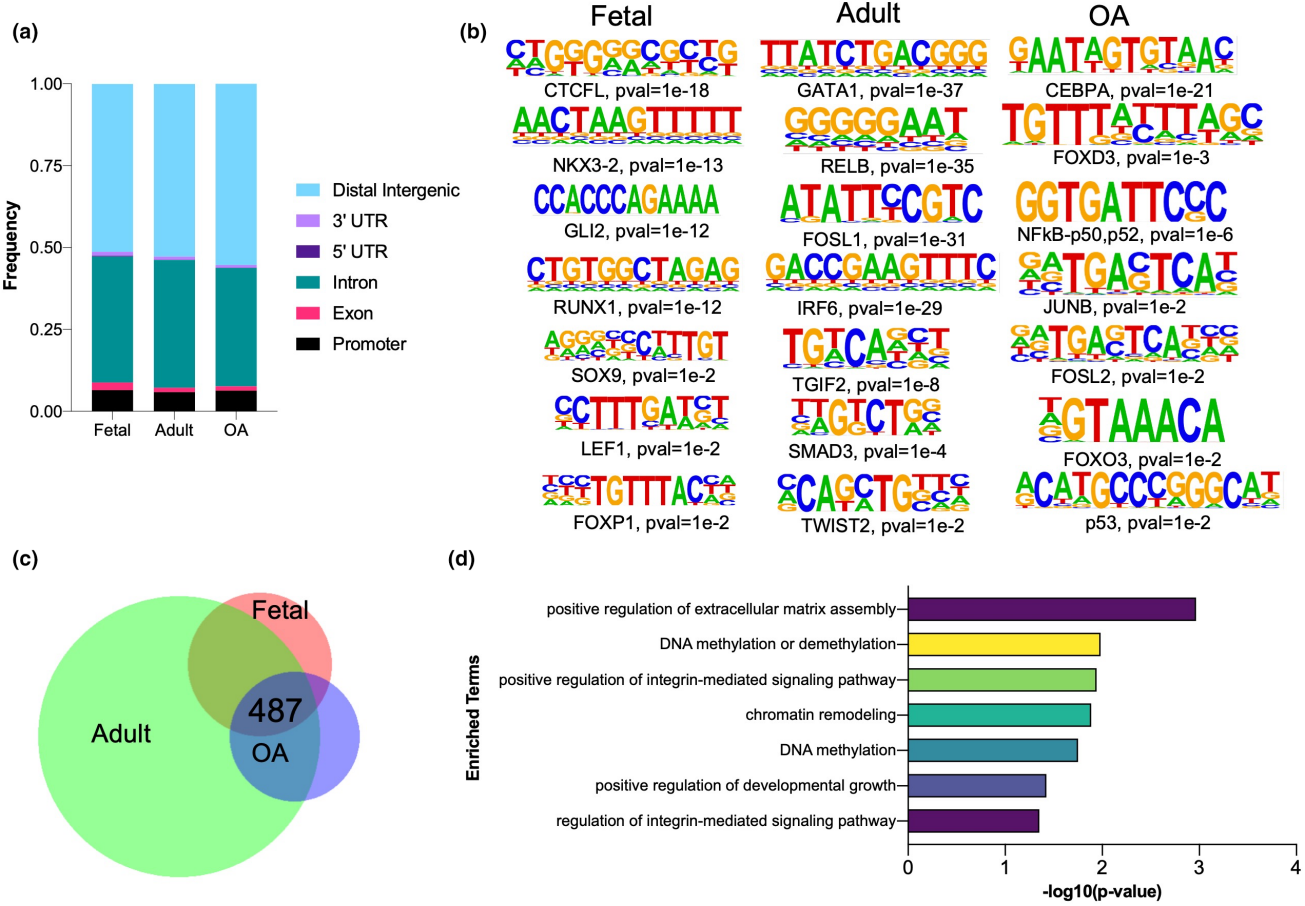
STAT3 binding targets during chondrocyte development and disease. (a) Bar graph showing the distribution of genomic features for peaks in fetal, adult and osteoarthritic (OA) chondrocytes. (b) DNA motif enrichment analysis for putative STAT3 binding targets. Binomial distribution was used to score motifs. (c) Venn diagram showing the overlap between putative STAT3 targets in fetal, adult and OA chondrocytes. (d) Gene ontology enrichment analysis for genes obtained from (c).

To gain further insight into the context‐specific putative targets of STAT3, we performed Cleavage Under Targets and Release Using Nuclease (CUT&RUN) profiling on freshly isolated non‐cultured fetal (*n* = 4), adult (*n* = 4), and OA chondrocytes (*n* = 8) (Figure S7). Most of the STAT3‐binding sites were located in the distal intergenic regions, suggesting STAT3 might regulate the expression of its putative targets by binding to distal regulatory elements (Figure 4a). Interestingly, epigenetic regulation mediated by STAT3 via binding to intergenic regions has been reported previously (Tripathi et al., 2017). Subsequently, DNA motif enrichment analysis for putative STAT3 targets was performed (Figure 4b). For fetal chondrocytes, we obtained motifs for several well‐validated and important transcription factors known to modulate early development, including SOX9 (Lefebvre, 2019). A similar analysis for adult chondrocytes showed enrichment for GATA1, RELB, IRF6, SMAD3, TWIST2, and other binding motifs. Although the role of these genes in chondrocytes is not completely clear, these transcription factors are known to be essential for differentiation and lineage commitment in different cell types (Alvisi et al., 2020; Kitajima et al., 2006; Natsumeda et al., 2021). For OA chondrocytes, we obtained a DNA motif for NF–kB, which is a well‐known transcription factor that mediates inflammation. Recently, Wang et al. have demonstrated that STAT3 can accelerate the onset of OA through the NF–kB signaling pathway (Wang et al., 2020). Other transcription factors that might regulate OA via co‐binding to STAT3 include JUNB, FOSL2, and FOXO3, mostly known for their role in inflammation (Fan et al., 2010; Hneino et al., 2012; Renoux et al., 2020; Thomsen et al., 2013). We next overlapped the putative targets obtained from fetal, adult, and OA chondrocytes and determined that 487 putative targets were shared in development and disease (Figure 4c). Most of these genes are known to be involved in extracellular matrix organization and skeletal system development (Muir, 1995) (Figure 4d). Interestingly, we observed that these genes are also involved in DNA methylation and chromatin remodeling indicating that STAT3 mediates its context‐specific role, at least in part, via epigenetic regulation (Figure 4d). Thus, combinatorial analysis of these data provides critical insight into the multipotential, and context‐specific mode of regulation exhibited by STAT3 during development and disease.

### 2.6 STAT3 regulates the levels of DNMT3B in human chondrocytes

**FIGURE 5:**
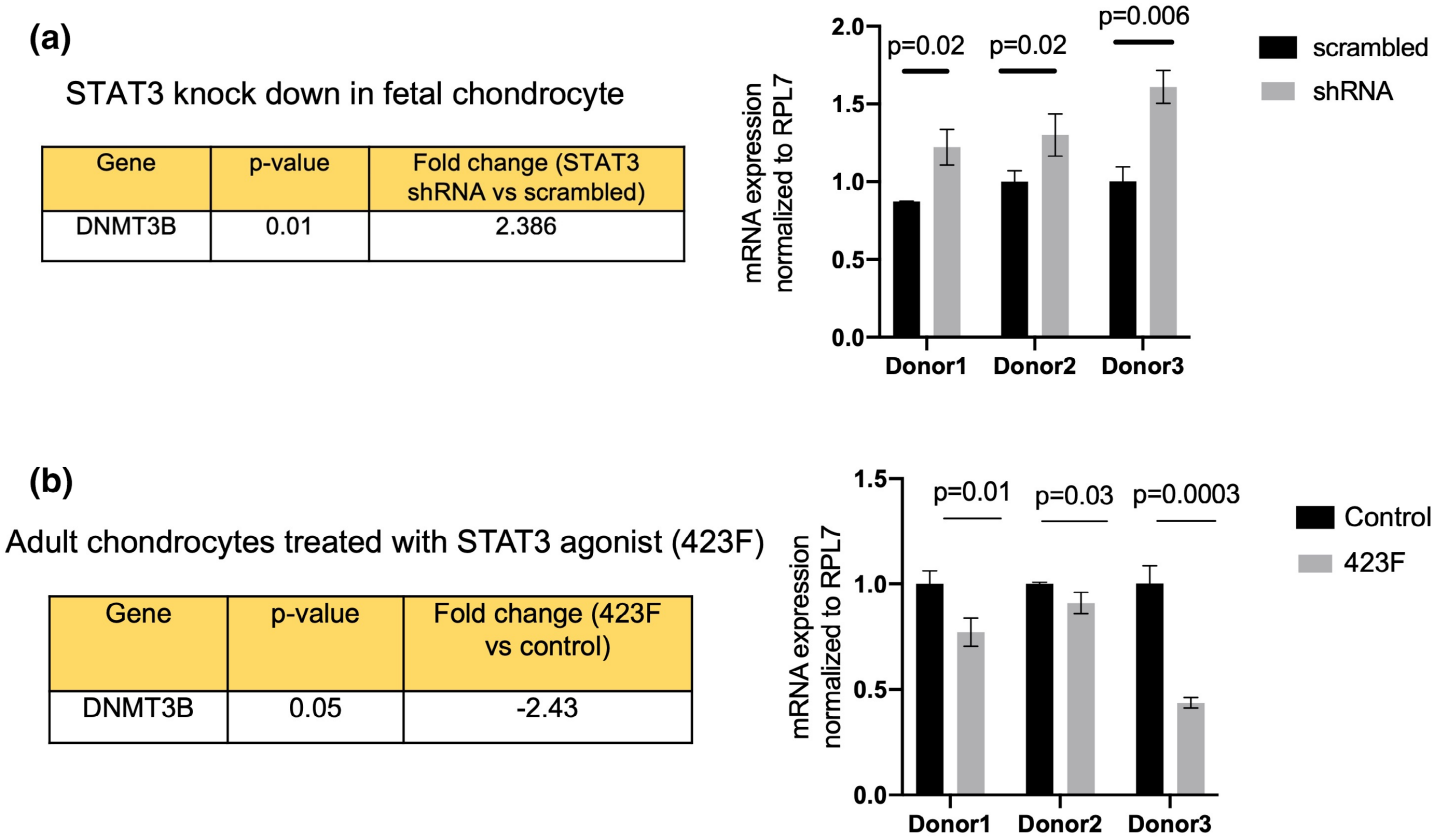
STAT3 regulates the levels of DNMT3B in human chondrocytes. (a) STAT3 knock down in fetal chondrocyte leads to transcriptional activation of DNMT3B. (b) Pharmacological activation of STAT3 in adult chondrocyte leads to transcriptional repression of DNMT3B. Statistical analysis was performed using two‐tailed Student's *t* test in GraphPad prism 9.0 and *p* < 0.05 was considered as statistically significant. Mean with standard deviation is plotted.

Our study suggests that the context‐specific role of STAT3 in chondrocyte development and disease is mediated by DNA methylation. DNA methyltransferases (DNMTs) are known to catalyze DNA methylation, of which DNMT3B serves as a *de novo* DNMT by establishing new methylation pattern and is known to play an important role in cartilage development (Fernandez‐Tajes et al., 2014). Interestingly, DNMT3B was found to be among a few putative STAT3 binding targets overlapping between development and disease (Figure 4c,d). Upon analyzing the bulk RNA‐sequencing data obtained from STAT3 knocked down fetal chondrocytes (Liu, Lin, Li, Lu, Geng, Zhang, et al., 2022), we observed an increase in expression of DNMT3B (fold change = 2.386, *p* = 0.01), indicating that STAT3 binding represses transcription of DNMT3B, which is reversed upon STAT3 knock down (Figure 5a). Also, pharmacological activation of STAT3 signaling in adult chondrocytes (Shkhyan et al., 2018) results in significant downregulation of DNMT3B (fold change = −2.43, *p* = 0.05) (Figure 5b) accompanied with a significant decrease in DNA methylation. We further validated this observation using qPCR data from *in vitro* experiments with STAT3 knocked down fetal chondrocytes as well as pharmacological activation of STAT3 in adult chondrocytes. Hence, we elucidate a model (Figure S8) for STAT3‐mediated DNA methylation regulation. We propose that the global DNA methylation changes induced either upon STAT3 knock down in fetal chondrocytes or its pharmacological activation in adult chondrocytes might be mediated by DNMT3B, thereby leading to alterations in epigenetic age.

### 2.7 Osteoarthritic chondrocytes possess an immature phenotype

**FIGURE 6:**
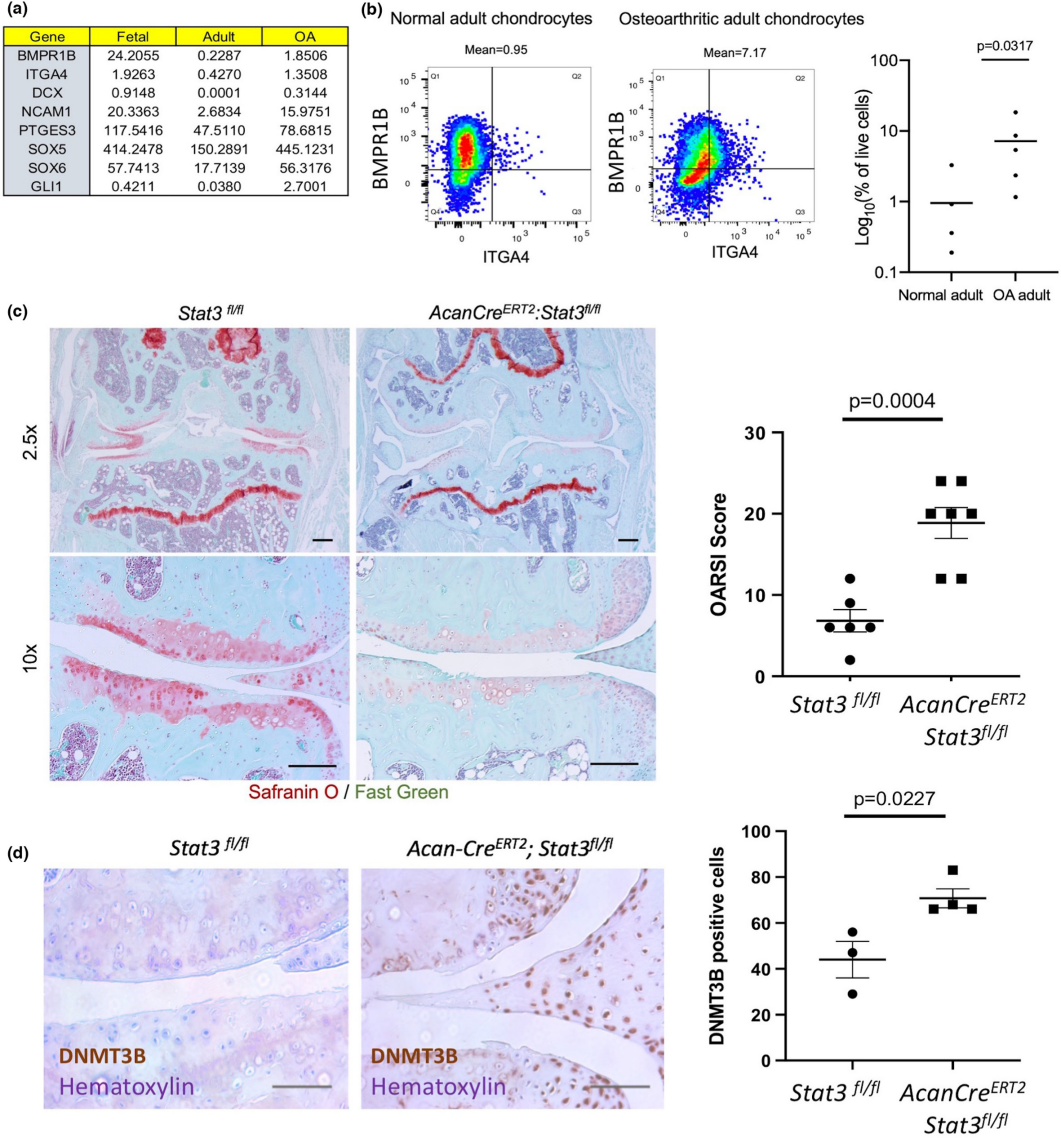
Functional outcomes of OA chondrocytes. (a) Gene expression levels for genes involved in maintaining immature and proliferative phenotype of chondrocytes. (b) Representative flow cytometry plots and quantification of ITGA4 and BMPR1B expression in normal adult and OA adult chondrocytes. *p*‐values were calculated using an unpaired Mann–Whitney test. (c) Histological staining and quantitative assessment of cartilage degradation of mice knee joints 8 weeks after DMM surgery. Safranin O delineates proteoglycans in the articular cartilage (pink). Post‐traumatic OA mouse model shows accelerated progression of OA upon *Stat3* deletion as evident by the OARSI score compared to control mice. Magnification = 2.5X and 10X. Scale bars represent 400 μm. Horizontal lines with bars show the mean ± SD. *n* = 6–7. (d) Histological staining and quantitative assessment of DNMT3B in PTOA mouse model shows increased expression of DNMT3B upon *Stat3* deletion. Magnification = 20X. Scale bars represent 50 μm. Horizontal lines with bars show the mean ± SD. DNMT3B positive cells per 20X field on medial tibial articular cartilage have been quantified.

Based on the current study and previous studies from our lab, it is evident that higher expression of pSTAT3 confers an immature and proliferative phenotype in fetal and adult chondrocytes (Shkhyan et al., 2018). This prompted us to explore if higher expression of STAT3 in OA chondrocytes (Figure S6) induces a similar fetal‐like immature program as an intrinsic attempt to regenerate the lost tissue in response to injury. We thus investigated the gene expression levels of immature/proliferative genes (including ITGA4, BMPR1B, SOX5, 6, GLI1, NCAM1, DCX, and others) (Ferguson et al., 2018) across developing human fetal chondrocytes, normal adult chondrocytes, and OA adult chondrocytes. Surprisingly, we observed a higher expression of these genes in fetal and OA adult chondrocytes as compared to normal adult chondrocytes (Figure 6a). It has been previously shown by our group that ITGA4^+^BMPR1B^+^ cells confer an immature phenotype to adult chondrocytes (Ferguson et al., 2018). Also, this population of cells shows higher basal levels of pSTAT3 expression as well as high levels of chondroprogenitor genes such as SOX9 and GLI1 (Ferguson et al., 2018). This impelled us to investigate the presence of a similar immature and proliferative population marked by ITGA4^+^BMPR1B^+^ in OA adult chondrocytes. Flow cytometry analyses of ITGA4 and BMPR1B expression in normal adult (*n* = 5) and OA adult chondrocytes (*n* = 5) showed an enriched population of ITGA4^+^BMPR1B^+^ cells in OA samples relative to adult chondrocytes (Figure 6b), further supporting the gene expression levels. To gain further insight into the role of STAT3 in the context of disease, *in vivo* studies were performed in a post‐traumatic OA (PTOA) mouse model. OA was surgically induced by destabilization of the medial meniscus (DMM) in the left knee joint of the mice as described previously (Glasson et al., 2007). To assess the function of STAT3 *in vivo*, we crossed Stat3^fl/fl^ mice with the Acan‐Cre^ERT2^ strain, enabling tamoxifen‐inducible deletion of *Stat3* in chondrocytes with a single allele of Acan‐Cre^ERT2^. DMM surgery was performed 1 week after the second dose of tamoxifen administration to eliminate any possibility of Cre recombination after injury. Upon conditional *Stat3* deletion in chondrocytes driven by the inducible *Acan‐Cre*, the PTOA mouse model showed a higher OARSI score indicative of a more prominent OA progression (Figure 6c). As a further confirmation of STAT3‐mediated DNA methylation regulation, we evaluated the expression of DNMT3B upon *Stat3* deletion in this model. In addition to a more prominent OA progression, a significant increase in DNMT3B expression was observed upon *Stat3* deletion (Figure 6d); the data supported our *in vitro* findings that expression of DNMT3B, a direct STAT3 target, is inversely correlated to STAT3 levels in articular chondrocytes. Altogether, this data suggest that OA chondrocytes may undergo epigenetic‐age reversal in the attempt to activate a progenitor‐like phenotype driven by elevated STAT3 expression.

## 3 DISCUSSION

Articular chondrocytes undergo functional changes and their regenerative potential declines with age. Development and differentiation of chondrocytes is governed by cell‐specific gene expression patterns, which are established and reinforced by DNA methylation (Singh et al., 2021). STAT3, a key transcriptional factor, has been previously known to be involved in regulating stemness, development, and regeneration of tissues and organs via epigenetic mechanisms (Burdon et al., 2002; Moresi et al., 2019). We have previously reported that STAT3 is highly expressed in anabolic proliferative fetal chondrocytes (Shkhyan et al., 2018) and is involved in chondrocyte development. Recently, we have also shown that early postnatal STAT3 deletion in chondrocytes in mice leads to suppression of chondrocyte proliferation and eventually, degradation of the growth plate evident by 3 months of age (Liu, Lin, Li, Lu, Geng, Zhang, & Evseenko, 2022). On the other hand, overexpression of STAT3 in postnatal chondrocytes in mice resulted in their hyperproliferation (Liu, Lin, Li, Lu, Geng, Zhang, & Evseenko, 2022).

Although the role of STAT3 in gene regulation is widely studied, the direct role of STAT3 in regulating the epigenetic status of cartilage cells has not been reported. The critical role of STAT3 in development intrigued us to understand its effect in modulating DNA methylation. In this study, we show that pharmacological activation of STAT3 signaling in normal aged adult chondrocytes decreases their biological age *in vitro*. We also show that genetic ablation of STAT3 in fetal chondrocytes, induces a global hypermethylation, in accordance with its role in maintaining an immature phenotype in chondrocytes. In addition, transcriptional targets of STAT3 in chondrocytes have not been determined previously. Here, we observe that putative binding targets of STAT3 in fetal, normal adult and OA chondrocytes are associated with distinct signaling pathways.

Our data suggest that the context‐specific role of STAT3 in development and disease may be explained in part by its regulation of DNA methylation in chondrocytes via DNMT3B, which plays an important role in early cartilage development (Fernandez‐Tajes et al., 2014; Sanchez‐Fernandez et al., 2020). Here we show that the STAT3 expression level determines the transcriptional activation/repression of DNMT3B in a context‐specific manner. Although we provide a probable hypothesis that might help in understanding the presumptive mechanism for STAT3 mediated DNA methylation in human chondrocytes via DNMT3B, it needs further detailed investigation.

An anabolic role of STAT3 in developing chondrocytes as well as the important role of this transcription factor in stem cells and regeneration in various tissues has been studied (Nakao et al., 2020). Transient and highly calibrated STAT3 activation is required for regeneration after injury, but chronic activation of this pathway by inflammatory processes may lead to fibrosis and degeneration (Pickert et al., 2009). Thus, it was plausible to predict the same regulatory pattern in cartilage tissue. Activation of STAT3 in OA chondrocytes is likely to indicate an intrinsic attempt of the tissue to regenerate by enriching for immature ITGA4^+^BMPR1B^+^ cells highly expressing STAT3 and also other progenitor markers such as SOX9 (Ferguson et al., 2018). Since STAT3 signaling is already highly upregulated in OA chondrocytes, modulation of the intrinsic levels of STAT3 signaling therapeutically may be challenging due to a multifaceted nature of this pathway. Our previous studies have shown that in two PTOA osteochondral defect models in rat, STAT3 activation was beneficial to induce proliferation of cartilage cells in the injured joint and induce cartilage repair (Shkhyan et al., 2018). Unfortunately, this model represents an acute injury as cartilage defects heal within 2–4 weeks in rats unlike in chronic human OA. In the current study, we also show that *Stat3* deletion in a PTOA mouse model leads to augmentation in severity of the disease. Albeit transient STAT3 activation under controlled conditions may be beneficial, many risks are involved in its constitutive activation as evidenced by myriad of diseases resulting in degeneration, fibrosis, and cancer development of various organs (Catlett‐Falcone et al., 1999). However, complete inhibition of this pathway may also be detrimental since, as previously mentioned, STAT3 plays crucial roles in many normal biological processes such as tissue regeneration. Thus, modulatory drugs which do not interfere with the intrinsic levels of STAT3 activation in OA are superior to complete inhibition.

The current study has a few limitations. OA is a heterogenous chronic disease and is characterized by multiple functional stage‐specific changes. Although the current study sheds some light on the context‐specific functions of STAT3, more patients need to be analyzed to establish a definitive, stage‐specific role of this transcription factor in the onset and progression of OA. It is also likely that different subsets of OA chondrocytes may have different DNA methylation signature that were not explored in this study. Other limitations for this study include exploring other modes of epigenetic regulation exhibited by STAT3 (i.e., via histone methylation or acetylation) and using an uniform digestion time across chondrocyte samples to minimize differential effects. DNA methylation profiling and an epigenetic clock for OA chondrocytes might help us better understand the epigenetic changes in disease. Moreover, further experiments with larger cohort of human donors, delineating the direct physiological effect of changed methylation pattern on chondrocyte age will strengthen the current study.

## 4 CONCLUSIONS

In summary, the data presented here serve as a foundation to understand the complex regulation of the epigenome across human chondrocyte ontogeny. Furthermore, these studies provide strong evidence for the crucial context‐specific role of STAT3 in regulating chondrocyte development and disease in addition to suggesting that activation of STAT3 signaling may reduce epigenetic age in adult cells and may be part of the regenerative mechanism induced by inflammatory processes. However, the role of STAT3 is context‐specific as uncontrolled activation is unfavorable.

This study elucidates a pivotal mechanism that, if targeted, may preclude the underlying dysfunction in age‐associated processes preventing deleterious changes in cartilage and inhibiting consequential systemic decline of joint function. The novel epigenetic clock presented here will help researchers to capture pivotal aspects of biological age in adult chondrocytes. We anticipate this work will shed light towards chondrocyte aging with newer perspectives for clinical development of novel agents for joint preservation. Most importantly, the groundbreaking data presented here may be applied to a multitude of age‐related diseases beyond diseases of the joint.

### 4.1 Experimental procedures

Detailed description of all experimental procedure is provided in Supplementary Material.

### 4.2 DNA methylation data

The Illumina Infinium Methylation EPIC BeadChip array was used to perform DNA methylation profiling.

### 4.3 DNA methylation age and epigenetic clock

The chondrocyte clock was developed using both novel and existing methylation data from chondrocytes, cartilage, and bone. The age was regressed on DNA methylation levels using elastic net regression as implemented in the R function glmnet.

### 4.4 Cleavage under targets and release using nuclease (CUT & RUN)

*In situ* chromatin profiling using CUT & RUN was performed for fetal, adult and OA chondrocytes.

## AUTHOR CONTRIBUTIONS

AS, SH, KL, and DE conceptualized the study and interpreted the data. AS, NQL, JM JT, SL, RS, YL, JL, YO, HT, FB, and LT performed experiments. AS, JE, and SH analyzed the data. NS, JE, SH, and DE provided conceptual insight. AS, RS, and DE wrote the manuscript. All the authors read and approved the manuscript.

## FUNDING INFORMATION

This work was supported by the National Institutes of Health grant R01AR071734 (DE) and the National Institutes of Health grant R01AG058624 (DE), Department of Defense grant W81XWH‐13‐1‐0465 (DE), California Institute for Regenerative Medicine grant TRAN1‐09288 (DE).

## CONFLICT OF INTEREST

The authors declare that they have no conflict of interests.

## Supporting information


TableS1

Supinfo

## Data Availability

All data is deposited in GEO and is available under the accession number GSE181356.
