# Supplementary material for: STAT3 promotes a youthful epigenetic state in articular chondrocytes
Source: Aging Cell. 2023 Jan 13;22(2):e13773. doi: 10.1111/acel.13773 (PMC9924946; doi:10.1111/acel.13773)
Supplement: Supplementary file 2 — Supinfo [file ACEL-22-e13773-s001.docx]

**Supplementary Experimental procedures**

**Chondrocyte sample collection**

Human fetal chondrocytes (14wks-19wks) from the interzone were isolated as previously described (Wu et al., 2013) from de-identified material from anonymous donors following informed consent; as no means of associating biospecimens with donors was possible, this does not meet the definition of human subjects research and is therefore exempt from ethical review. Adult healthy human primary (21yrs-87yrs, males and females) cartilage specimens were obtained from National Disease Research Interchange (NDRI). Chondrocytes were isolated from the femoral condyle of the knee joint. Human OA samples were obtained from NDRI and Dr. Jay Lieberman (protocol HS-16-00449 approved by the USC IRB Committee). These knee OA samples (55yrs-81yrs, males and females) were dissected from the tibial and femoral articular cartilage, mostly from visually intact regions available in the specimen. All patients had 3–4 on the Kellgren–Lawrence grading scale for OA. All donated material was anonymous and carried no personal identiﬁers. Samples were processed following previously established protocols.

Briefly, primary tissues were manually cut into small pieces and digested for 4–16 h at 37 °C with mild agitation in digestion media consisting of DMEM (Corning) with 10% FBS (Sigma), 1 mg/mL dispase (Gibco), 1 mg/mL type 2 collagenase (Worthington), 10 –µg/mL gentamycin (Teknova) and 100 µg/mL primocin (Invivogen). Samples were subjected to sequencing after the FACS-based purification of viable cells. All analyses were conducted on non-cultured cells unless additionally specified to avoid cell de-differentiation or any other phenotypic changes.

**Genomic DNA extraction**

Genomic DNA was extracted using QIAGEN DNeasy® Tissue kit or QIAamp® DNA Micro Kit depending on the starting number of cells. For DNeasy® Blood or Tissue kit samples were first lysed using Proteinase K. Lysate was loaded into the DNeasy Mini spin column and centrifuged to selectively bind DNA to the DNeasy membrane as contaminants pass through. Subsequent washing steps remove remaining contaminants and enzyme inhibitors. For QIAamp® DNA Micro Kit samples were lysed under high denaturing conditions at elevated temperatures in the presence of Proteinase K and Buffer ATL. Buffer AL was added to lysates followed by loading into QIAamp MinElute column and centrifugation. Residual contaminants or inhibitors are washed off using first Buffer AW1 and then Buffer AW2. Purified genomic DNA from either kit was eluted in water and quantified by Nanodrop confirming for high 260/280 purity ratio.

**DNA methylation data**

The Illumina Infinium MethylationEPIC BeadChip array was used to perform DNA methylation profiling. This platform measures bisulfite conversion–based, single-CpG-resolution DNA methylation levels at 866,836 CpG sites in the human genome. Methylation levels are quantified by β values which is the ratio of intensities between methylated (signal A) and un-methylated (signal B) alleles. Specifically, the β value is calculated from the intensity of the methylated (M corresponding to signal A) and un-methylated (U corresponding to signal B) alleles, as the ratio of fluorescent signals β = Max(M,0)/[Max(M,0) + Max(U,0) + 100]. Thus, β values range from 0 (completely un-methylated) to 1 (completely methylated) (Dunning et al., 2008).

**Analysis of Infinium EPIC methylation data**

The R package “minfi” was used for analysis of the data (Aryee et al., 2014; Fortin et al., 2017). Raw IDAT files were read and preprocessed and probes with high detection p-value (p-value>0.05) and potential SNP contamination were filtered. Normalization of data was done using the preprocessFunnorm function to generate Beta values per probe. Beta values provide the percentage of CpG methylation per probe with 0 being unmethylated and 1 fully methylated. Differentially methylated probes were identified by dmpFinder in logistic regression mode for appropriate contrasts followed by statistical analysis using an empirical Bayes method and then filtered by significance threshold (p-value<0.05, F-test). Annotation of probes was performed with the R package ‘IlluminaHumanMethylationEPICanno.ilm10b4.hg19’ version 0.6.0 for hg19 genome build. Gene ontology enrichment analysis for the nearest genes annotated to the peaks was determined by Enrichr (Chen et al., 2013). Genes expressed in the respective samples were used as the background genes. For EWAS approach (Horvath et al., 2012; Rakyan et al., 2010; Teschendorff et al., 2010), the DNA methylation changes were examined for association with chondrocyte age using the function "standardScreeningNumericTrait" from the "WGCNA" R package (Langfelder & Horvath, 2008).

**DNA methylation age and Epigenetic clock**

The chondrocyte clock was developed using both novel and existing methylation data from chondrocytes, cartilage and bone. The age was regressed on DNA methylation levels using elastic net regression as implemented in the R function glmnet (Friedman et al., 2010). The alpha parameter of glmnet was chosen as 0.5 (midpoint between Ridge and Lasso type regression) and the lambda value was chosen using cross-validation on the training dataset. A10 fold internal cross-validation (cv.glmnet) was used on the training data set to determine optimal penalty parameters. We performed a cross-validation scheme for arriving at unbiased (or at least less biased) estimates of the accuracy of the different DNAm based age estimators. For validation of the clocks, we used leave-one-out LOO cross-validation (LOOCV) in which one sample was left out of the regression, then predicted the age for the remaining samples and iterated this process over all samples.

**ChromHMM analysis**

We conducted ChromHMM (Ernst & Kellis, 2012) chromatin state enrichment analysis with chromatin state annotations from Fetal 17 weeks and adult chondrocytes tissues using a previously defined 12-state model (Ferguson et al., 2018). Hypermethylated and hypomethylated age-correlated CpGs were determined by EWAS as mentioned previously. Using the OverlapEnrichment command of ChromHMM we computed the enrichment for the coordinates set of hypermethylated and hypomethylated age-correlated CpGs. We did the same for the coordinates of all CpGs on the array, and then divided the hypermethylated and hypomethylated age-correlated CpG enrichment values by these enrichment values to obtain the enrichment relative to the array background.

**Cell culture and treatments**

Only early passages of fetal and adult chondrocytes (P0) were used for experimentation to avoid de-differentiation and loss of cartilage phenotype(Wu et al., 2014). Fetal and adult chondrocytes were cultured in DMEM F12 medium containing 10% (vol/vol) fetal bovine serum and 1% Penicillin-Streptomycin (vol/vol) at 37 °C in a humidified atmosphere of 95% air and 5% CO_2_. Media was replenished with DMEM F12 medium containing 1% (vol/vol) fetal bovine serum and 1% Penicillin-Streptomycin (vol/vol) once treatments were added.

Genetic ablation of STAT3 in fetal chondrocytes was performed using a SMARTvector Inducible Lentiviral shRNA (Dharmacon) reporter-based approach as described previously (Liu et al., 2022a). Briefly, P0 fetal chondrocytes were transduced with doxycycline inducible STAT3 hEF1a-TurboRFP shRNA or scrambled lentiviral particles (Dharmacon). After 24hrs, transduced chondrocytes were treated with doxycycline; fresh doxycycline was added every 48hrs. After 4 weeks of treatment, transduced cells were sorted for viable cells with RFP fluorescence.

Aged adult chondrocytes (55yrs-87yrs) were treated with or without a modified form of the small molecule STAT3 agonist, RCGD 423F N-(4-Fluorophenyl)-4-phenyl-2-thiazolamine; synthesized and provided by J-STAR Research at 10µM for 2weeks.

**ATAC-sequencing and data analysis**

ATAC-sequencing was performed on P0 fetal chondrocytes transduced with doxycycline inducible STAT3 shRNA (n=3) or scrambled (n=3) lentiviral particles (Dharmacon). Samples were washed, lysed followed by nuclei tagmentation and adapter ligation using the Diagenode ATAC-seq kit. Transposed DNA fragments were amplified using the HiFi PCR Master Mix with regular forward and reverse barcoded primers. The final size-selected product was purified, and quality checked on 2100 Bioanalyzer (Agilent). Sequencing was performed on Illumina NovaSeq SP with paired-end 50 base pair reads. The initial quality of the raw fastq files were checked using FastQC (<https://www.bioinformatics.babraham.ac.uk/projects/fastqc/>). Reads were trimmed using Trimmomatic (Bolger et al., 2014) in paired-end mode. Trimmed reads were aligned to human genome build hg19 using bowtie2. PCR duplicates were removed from the aligned reads followed by sorting and indexing of the bam files by Picard’s MarkDuplicates function (http://broadinstitute.github.io/picard). Tn5 transposase binds as a dimer and inserts two adapters separated by 9 bp (Adey et al., 2010). Thus, prior to peak calling, reads were shifted by +4 bp for the positive strand and -5 bp for the negative strand. Significant peaks (p-value<0.05) were called using MACS2 (Zhang et al., 2008) and annotated using ChIPseeker (Yu et al., 2015).

**Seahorse Real-Time ATP Rate Assay**

ATP production rate assay for STAT3 ablated human fetal chondrocytes was performed using XF Real-Time ATP Rate Assay kit (Agilent). Cells were seeded in triplicates on poly-D-lysine coated XF cell culture microplate in assay medium (XF DMEM medium pH 7.4 supplemented with 10mM glucose, 2mM glutamine and 1mM pyruvate) followed by incubation at 37 °C in a non-CO_2_ incubator for 1hr. ATP measurements were recorded followed by Oligomycin injection in port A at 1.5μM final concentration, and Rotenone/Antimycin injection in port B at 0.5μM final concentration. Cytation 5 Imaging system was used to count cells in each well and normalize the data. Data analysis was performed using Report Generators software (Agilent).

**Cleavage Under Targets and Release Using Nuclease (CUT&RUN)**

*In situ* chromatin profiling using CUT & RUN was performed according to Skene et al. (Skene et al., 2018). Briefly samples were FACS sorted using DAPI to select live cells and 10,000 cells were collected in 10%FBS-PBS media. Cell nuclei were immobilized on Concanavalin A beads after washing. pSTAT3 (Tyr705, D3A7,9145, Cell signaling technology) or normal rabbit IgG antibodies (3900,Cell signaling technology) were incubated with the nuclei overnight in the presence of 0.02% digitonin at 4°. The next day, 700ng/mL of proteinA-micrococcal nuclease (pA-Mnase purified in house with vector from Addgene 86973 (Schmid et al., 2004)) were incubated with the nuclei at 4 degrees for an hour. After washing, the tubes were placed in heat blocks on ice set to 0 degrees, CaCl_2_ (1mM) was added and incubated for 30 min before 2X Stop buffer containing EDTA was added. DNA was extracted using Qiagen DNA isolation kit according to manufacturer’s protocol Purified DNA was quantified in Qubit and Bioanalyzer (2100) traces using D5000 high sensitivity chip were run to determine the size of the cleaved products. UMI-coded libraries were generated using Swift Biosciences-ACCEL-NGS® 2S PLUS DNA LIBRARY KITS according to manufacturer’s protocol. Pair-end (75bp) Illumina sequencing was performed on the UMI-coded and amplified libraries using NextSeq platform.

**CUT&RUN data analysis**

UMI-tools (Smith et al., 2017) ‘extract’ function was used to remove UMIs from each read of the raw fastq files and append them to the read name. The initial quality of the raw fastq files were checked using FastQC (<https://www.bioinformatics.babraham.ac.uk/projects/fastqc/>). Reads were trimmed using Cutadapt v2.10 (Martin, 2011) in paired-end mode. Trimmed reads were aligned to human genome build hg19 using bowtie2. Next, aligned reads were deduplicated and PCR duplicates were removed by UMI-tools ‘dedup’ function followed by sorting and indexing of the deduplicated bam files by SAMtools v1.11 (Li et al., 2009). Pearson correlation plots were generated using deepTools suite v3.5.0 (Ramirez et al., 2016). Significant peaks (p-value<0.05) were called from the deduplicated reads using MACS2 (Zhang et al., 2008) and annotated using the R package ChIPseeker (Yu et al., 2015). Subsequently, peak files were used to determine enriched motifs using HOMER v4.11.1 (Heinz et al., 2010). Two-way and three-way Venn diagrams were generated using BioVenn (Hulsen et al., 2008). Gene ontology enrichment analysis for the nearest genes annotated to the peaks was determined by Enrichr (Chen et al., 2013). Genes expressed in the respective samples were used as the background genes.

**RNA extraction and quantitative Real-Time PCR**

Total RNA was extracted from live sorted fetal chondrocytes transduced with STAT3 shRNA or scrambled and STAT3 agonist treated adult chondrocytes using the RNeasy Mini Kit (Qiagen). 500 ng of RNA was reverse transcribed using the Maxima First Strand cDNA Synthesis Kit (Thermo Fisher). Power SYBR Green (Applied Biosystems) RT-PCR amplification and detection was performed using an Applied Biosystems Step One Plus Real-Time PCR machine. The comparative Ct method for relative quantification (2^-ΔΔCt^) was used to quantitate gene expression, where results were normalized to RPL7 (ribosomal protein L7). Primer sequences are available upon request. Results were analyzed using 2-tailed Student’s t test in GraphPad Prism 9.0.

**Western blot analysis**

Osteoarthritic chondrocytes were lysed in RIPA Lysis and Extraction Buffer (Pierce) containing protease inhibitors (Pierce) followed by sonication with a 15-second pulse at a power output of 2 using the VirSonic 100 (SP Industries Company). Protein concentrations were determined by BCA protein assay (Pierce) and boiled for 5 minutes with Laemmli Sample Buffer (Bio-Rad, Hercules, CA). Proteins were separated on acrylamide gels and analyzed by Western blot using primary antibodies: anti-pSTAT3 (9145) and anti-Histone H3 (9515; all from Cell Signaling). Histone H3 antibody was used as a loading control. Proteins were resolved with SDS-PAGE utilizing 4–15% Mini-PROTEAN TGX Precast Gels and transferred to Trans-Blot Turbo Transfer Packs with a 0.2-µm pore-size nitrocellulose membrane. The SDS-PAGE running buffer, 4–15% Mini-PROTEAN TGX Precast Gels, Trans-Blot Turbo Transfer Packs with a 0.2-µm pore-size nitrocellulose membrane was purchased from Bio-Rad. Nitrocellulose membranes were blocked in 5% nonfat milk in 0.05% (v/v) Tween 20 (Corning). Membranes were then incubated with primary antibodies overnight. After washing in PBS containing 0.05% (v/v) Tween 20 (PBST), membranes were incubated with secondary antibodies (31460 and 31430, Thermo Scientific). After washing, development was performed with the Clarity Western ECL Blotting Substrate (Bio-Rad).

**Flow cytometry**

Flow cytometry analysis was performed on a BD FACSAria IIIu cell sorter. Cells were washed twice in 1-2% FBS and stained with DAPI for viability. For fetal chondrocytes transduced with STAT3 shRNA or scrambled, populations of interest were analyzed based on DAPI negativity expression and RFP expression followed by direct sorting into DMEM/F12 containing 10% FBS with 1% P/S/A. For P0 adult and osteoarthritic chondrocytes, populations of interest were analyzed based on DAPI negativity for live cells, BMPR1B – PE expression (R&D Systems), and ITGA4 – PECy7 (Biolegend) at 1 μL antibody/10^6^ cells. Flow cytometry data was analyzed using FlowJo software (BD).

**Mouse lines and breeding**

All procedures involving animals were approved by the Institutional Animal Care and Use Committee (IACUC) of USC. This study was compliant with all relevant ethical regulations regarding animal research. Mice with tamoxifen-regulated expression of the Cre recombinase under control of the chondrocyte-specific aggrecan (Acan) promotor (Acan-Cre^ERT2^) were purchased from JAX (strain 019148) (Henry et al., 2009) and used exclusively as heterozygotes (Acan-Cre^+/ERT2^). To delete Stat3, these mice were crossed with mice bearing floxed Stat3 alleles (Stat3^fl/fl^), also purchased from JAX (strain 016923) (Liu et al., 2022b). All animals were on a C57BL/6 background. Genotyping was performed according to protocols provided by JAX. 4- to 5-month-old mice received tamoxifen (100 µg per gram of body weight) in corn oil via intraperitoneal injection daily for 2 consecutive days. 1 week after the second dose of tamoxifen administration, DMM surgery was performed.

**Post traumatic osteoarthritis mouse model**

OA was surgically induced by destabilization of the medial meniscus (DMM) in the left knee joint of male mice (Glasson et al., 2007). Female mice were excluded from this study as they are known to have less severe OA after DMM (Ma et al., 2007). All the animal procedures were approved by IACUC. Surgeries were performed on the left knee joint under sterile conditions and general anesthesia. Mice (4-5 months old) were anesthetized using isoflurane at 2% with oxygen carrier via nasal inhalation using a nose cone. Animals received pre-operative doses of 5 mg/kg carprofen and buprenorphine SR (1 mg/kg) subcutaneously. The surgical area was shaved with an electric clipper and prepped with 10% Povidone-Iodine and 70% Isopropyl Alcohol.

A medial para-patellar ligament incision was performed with a #15 blade as previously described (Glasson et al., 2007) under a dissection microscope. The joint capsule was opened with micro-iris scissors. The medial meniscotibial ligament (MML) of the medial meniscus (MM) was exposed by blunt dissection. Sectioning of MML with micro-surgical scalpel, with the blade directed proximo-laterally, gave DMM. The MM was left intact. Following DMM, medial displacement of the MM occurs. The wound was carefully cleaned with sterile saline. The joint capsule was closed with a 7-0 Vicryl Suture (Ethicon, J488G) and skin was closed with a 5-0 Vicryl suture (Ethicon, J385H). Following surgeries, animals were allowed free cage activity. Carprofen was injected subcutaneously daily for two days after the surgery. The right knee joint was left intact. Safranin O/Fast Green staining was performed according to routine protocols and was used to perform Osteoarthritis Research Society International (OARSI) score as described (Pritzker et al., 2006).

**Histology and Immunohistochemistry**

Limb tissues were dissected and fixed in 10% formalin overnight. They were then decalcified with 14% EDTA, pH7.4, for 2 weeks at 4 °C. Decalcified tissue was then embedded in paraffin and cut at a thickness of 5 μm. Paraffin sections were deparaffinized and rehydrated by passage through xylene and 100, 95, and 70% ethanol. Tissue was then permeabilized with 0.5% PBST (0.5% Triton X-100 in PBS) for 20 min. Antigen retrieval was carried out in citrate buffer (pH 6.0) for 20 min at 95 °C. Inhibition of endogenous peroxidase activity was performed using 3% H2O2 for 10 min. Sections were blocked with 5% normal horse serum (NHS) in 0.5% PBST for 20 min and incubated overnight with primary antibody rabbit anti-DNMT3B (Thermo-Fisher Scientific, PA1-884; 1:100) in 5% NHS in 0.5% PBST at 4 °C. Slides were then incubated at room temperature (RT) for 20 min in secondary antibody-HRP (Vector Laboratories, MP-7401). The primary antibody was visualized by peroxidase substrate kit DAB (Vector Laboratories, SK-4100). Finally, slides were counterstained with Hematoxylin for 1 minute (Vector Laboratories, H-3401) and dehydrated to Xylene. Slides were viewed using a Zeiss Axio Imager.A2 Microscope and images were taken using Axiocam 105 color (chromogenic detection) camera with Zen 2 program. Standard microscope camera settings were used.

**References**

Adey, A., Morrison, H. G., Asan, Xun, X., Kitzman, J. O., Turner, E. H., Stackhouse, B., MacKenzie, A. P., Caruccio, N. C., Zhang, X., & Shendure, J. (2010). Rapid, low-input, low-bias construction of shotgun fragment libraries by high-density in vitro transposition. *Genome Biol, 11*(12), R119. <https://doi.org/10.1186/gb-2010-11-12-r119>

Aryee, M. J., Jaffe, A. E., Corrada-Bravo, H., Ladd-Acosta, C., Feinberg, A. P., Hansen, K. D., & Irizarry, R. A. (2014, May 15). Minfi: a flexible and comprehensive Bioconductor package for the analysis of Infinium DNA methylation microarrays. *Bioinformatics, 30*(10), 1363-1369. <https://doi.org/10.1093/bioinformatics/btu049>

Bolger, A. M., Lohse, M., & Usadel, B. (2014, Aug 1). Trimmomatic: a flexible trimmer for Illumina sequence data. *Bioinformatics, 30*(15), 2114-2120. <https://doi.org/10.1093/bioinformatics/btu170>

Chen, E. Y., Tan, C. M., Kou, Y., Duan, Q., Wang, Z., Meirelles, G. V., Clark, N. R., & Ma'ayan, A. (2013, Apr 15). Enrichr: interactive and collaborative HTML5 gene list enrichment analysis tool. *BMC bioinformatics, 14*, 128. <https://doi.org/10.1186/1471-2105-14-128>

Dunning, M. J., Barbosa-Morais, N. L., Lynch, A. G., Tavare, S., & Ritchie, M. E. (2008, Feb 6). Statistical issues in the analysis of Illumina data. *BMC Bioinformatics, 9*, 85. <https://doi.org/10.1186/1471-2105-9-85>

Ernst, J., & Kellis, M. (2012, Feb 28). ChromHMM: automating chromatin-state discovery and characterization. *Nat Methods, 9*(3), 215-216. <https://doi.org/10.1038/nmeth.1906>

Ferguson, G. B., Van Handel, B., Bay, M., Fiziev, P., Org, T., Lee, S., Shkhyan, R., Banks, N. W., Scheinberg, M., Wu, L., Saitta, B., Elphingstone, J., Larson, A. N., Riester, S. M., Pyle, A. D., Bernthal, N. M., Mikkola, H. K., Ernst, J., van Wijnen, A. J., Bonaguidi, M., & Evseenko, D. (2018, Sep 7). Mapping molecular landmarks of human skeletal ontogeny and pluripotent stem cell-derived articular chondrocytes. *Nat Commun, 9*(1), 3634. <https://doi.org/10.1038/s41467-018-05573-y>

Fortin, J. P., Triche, T. J., Jr., & Hansen, K. D. (2017, Feb 15). Preprocessing, normalization and integration of the Illumina HumanMethylationEPIC array with minfi. *Bioinformatics, 33*(4), 558-560. <https://doi.org/10.1093/bioinformatics/btw691>

Friedman, J., Hastie, T., & Tibshirani, R. (2010). Regularization Paths for Generalized Linear Models via Coordinate Descent. *J Stat Softw, 33*(1), 1-22. <https://www.ncbi.nlm.nih.gov/pubmed/20808728>

Glasson, S. S., Blanchet, T. J., & Morris, E. A. (2007, Sep). The surgical destabilization of the medial meniscus (DMM) model of osteoarthritis in the 129/SvEv mouse. *Osteoarthritis Cartilage, 15*(9), 1061-1069. <https://doi.org/10.1016/j.joca.2007.03.006>

Heinz, S., Benner, C., Spann, N., Bertolino, E., Lin, Y. C., Laslo, P., Cheng, J. X., Murre, C., Singh, H., & Glass, C. K. (2010, May 28). Simple combinations of lineage-determining transcription factors prime cis-regulatory elements required for macrophage and B cell identities. *Mol Cell, 38*(4), 576-589. <https://doi.org/10.1016/j.molcel.2010.05.004>

Henry, S. P., Jang, C. W., Deng, J. M., Zhang, Z., Behringer, R. R., & de Crombrugghe, B. (2009, Dec). Generation of aggrecan-CreERT2 knockin mice for inducible Cre activity in adult cartilage. *Genesis, 47*(12), 805-814. <https://doi.org/10.1002/dvg.20564>

Horvath, S., Zhang, Y., Langfelder, P., Kahn, R. S., Boks, M. P., van Eijk, K., van den Berg, L. H., & Ophoff, R. A. (2012, Oct 3). Aging effects on DNA methylation modules in human brain and blood tissue. *Genome Biol, 13*(10), R97. <https://doi.org/10.1186/gb-2012-13-10-r97>

Hulsen, T., de Vlieg, J., & Alkema, W. (2008, Oct 16). BioVenn - a web application for the comparison and visualization of biological lists using area-proportional Venn diagrams. *BMC Genomics, 9*, 488. <https://doi.org/10.1186/1471-2164-9-488>

Langfelder, P., & Horvath, S. (2008, Dec 29). WGCNA: an R package for weighted correlation network analysis. *BMC Bioinformatics, 9*, 559. <https://doi.org/10.1186/1471-2105-9-559>

Li, H., Handsaker, B., Wysoker, A., Fennell, T., Ruan, J., Homer, N., Marth, G., Abecasis, G., Durbin, R., & Genome Project Data Processing, S. (2009, Aug 15). The Sequence Alignment/Map format and SAMtools. *Bioinformatics, 25*(16), 2078-2079. <https://doi.org/10.1093/bioinformatics/btp352>

Liu, N. Q., Lin, Y., Li, L., Lu, J., Geng, D., Zhang, J., Jashashvili, T., Buser, Z., Magallanes, J., Tassey, J., Shkhyan, R., Sarkar, A., Lopez, N., Lee, S., Lee, Y., Wang, L., Petrigliano, F. A., Van Handel, B., Lyons, K., & Evseenko, D. (2022a, Jan 17). gp130/STAT3 signaling is required for homeostatic proliferation and anabolism in postnatal growth plate and articular chondrocytes. *Commun Biol, 5*(1), 64. <https://doi.org/10.1038/s42003-021-02944-y>

Liu, N. Q., Lin, Y. C., Li, L. L., Lu, J. X., Geng, D. W., Zhang, J. K., Jashashvili, T., Buser, Z., Magallanes, J., Tassey, J., Shkhyan, R., Sarkar, A., Lopez, N., Lee, S., Lee, Y., Wang, L. M., Petrigliano, F. A., Van Handel, B., Lyons, K., & Evseenko, D. (2022b, Jan 17). gp130/STAT3 signaling is required for homeostatic proliferation and anabolism in postnatal growth plate and articular chondrocytes. *Communications Biology, 5*(1). <https://doi.org/ARTN> 64

10.1038/s42003-021-02944-y

Ma, H. L., Blanchet, T. J., Peluso, D., Hopkins, B., Morris, E. A., & Glasson, S. S. (2007, Jun). Osteoarthritis severity is sex dependent in a surgical mouse model. *Osteoarthritis Cartilage, 15*(6), 695-700. <https://doi.org/10.1016/j.joca.2006.11.005>

Martin, M. (2011, 2011-05-02). Cutadapt removes adapter sequences from high-throughput sequencing reads [next generation sequencing; small RNA; microRNA; adapter removal]. *2011, 17*(1), 3. <https://doi.org/10.14806/ej.17.1.200>

Pritzker, K. P., Gay, S., Jimenez, S. A., Ostergaard, K., Pelletier, J. P., Revell, P. A., Salter, D., & van den Berg, W. B. (2006, Jan). Osteoarthritis cartilage histopathology: grading and staging. *Osteoarthritis Cartilage, 14*(1), 13-29. <https://doi.org/10.1016/j.joca.2005.07.014>

Rakyan, V. K., Down, T. A., Maslau, S., Andrew, T., Yang, T. P., Beyan, H., Whittaker, P., McCann, O. T., Finer, S., Valdes, A. M., Leslie, R. D., Deloukas, P., & Spector, T. D. (2010, Apr). Human aging-associated DNA hypermethylation occurs preferentially at bivalent chromatin domains. *Genome Res, 20*(4), 434-439. <https://doi.org/10.1101/gr.103101.109>

Ramirez, F., Ryan, D. P., Gruning, B., Bhardwaj, V., Kilpert, F., Richter, A. S., Heyne, S., Dundar, F., & Manke, T. (2016, Jul 8). deepTools2: a next generation web server for deep-sequencing data analysis. *Nucleic Acids Res, 44*(W1), W160-165. <https://doi.org/10.1093/nar/gkw257>

Schmid, M., Durussel, T., & Laemmli, U. K. (2004, Oct 8). ChIC and ChEC; genomic mapping of chromatin proteins. *Mol Cell, 16*(1), 147-157. <https://doi.org/10.1016/j.molcel.2004.09.007>

Skene, P. J., Henikoff, J. G., & Henikoff, S. (2018, May). Targeted in situ genome-wide profiling with high efficiency for low cell numbers. *Nat Protoc, 13*(5), 1006-1019. <https://doi.org/10.1038/nprot.2018.015>

Smith, T., Heger, A., & Sudbery, I. (2017, Mar). UMI-tools: modeling sequencing errors in Unique Molecular Identifiers to improve quantification accuracy. *Genome Res, 27*(3), 491-499. <https://doi.org/10.1101/gr.209601.116>

Teschendorff, A. E., Menon, U., Gentry-Maharaj, A., Ramus, S. J., Weisenberger, D. J., Shen, H., Campan, M., Noushmehr, H., Bell, C. G., Maxwell, A. P., Savage, D. A., Mueller-Holzner, E., Marth, C., Kocjan, G., Gayther, S. A., Jones, A., Beck, S., Wagner, W., Laird, P. W., Jacobs, I. J., & Widschwendter, M. (2010, Apr). Age-dependent DNA methylation of genes that are suppressed in stem cells is a hallmark of cancer. *Genome Res, 20*(4), 440-446. <https://doi.org/10.1101/gr.103606.109>

Wu, L., Bluguermann, C., Kyupelyan, L., Latour, B., Gonzalez, S., Shah, S., Galic, Z., Ge, S., Zhu, Y., Petrigliano, F. A., Nsair, A., Miriuka, S. G., Li, X., Lyons, K. M., Crooks, G. M., McAllister, D. R., Van Handel, B., Adams, J. S., & Evseenko, D. (2013). Human developmental chondrogenesis as a basis for engineering chondrocytes from pluripotent stem cells. *Stem Cell Reports, 1*(6), 575-589. <https://doi.org/10.1016/j.stemcr.2013.10.012>

Wu, L., Gonzalez, S., Shah, S., Kyupelyan, L., Petrigliano, F. A., McAllister, D. R., Adams, J. S., Karperien, M., Tuan, T. L., Benya, P. D., & Evseenko, D. (2014, Feb). Extracellular matrix domain formation as an indicator of chondrocyte dedifferentiation and hypertrophy. *Tissue Eng Part C Methods, 20*(2), 160-168. <https://doi.org/10.1089/ten.TEC.2013.0056>

Yu, G., Wang, L. G., & He, Q. Y. (2015, Jul 15). ChIPseeker: an R/Bioconductor package for ChIP peak annotation, comparison and visualization. *Bioinformatics, 31*(14), 2382-2383. <https://doi.org/10.1093/bioinformatics/btv145>

Zhang, Y., Liu, T., Meyer, C. A., Eeckhoute, J., Johnson, D. S., Bernstein, B. E., Nusbaum, C., Myers, R. M., Brown, M., Li, W., & Liu, X. S. (2008). Model-based analysis of ChIP-Seq (MACS). *Genome Biol, 9*(9), R137. <https://doi.org/10.1186/gb-2008-9-9-r137>

**
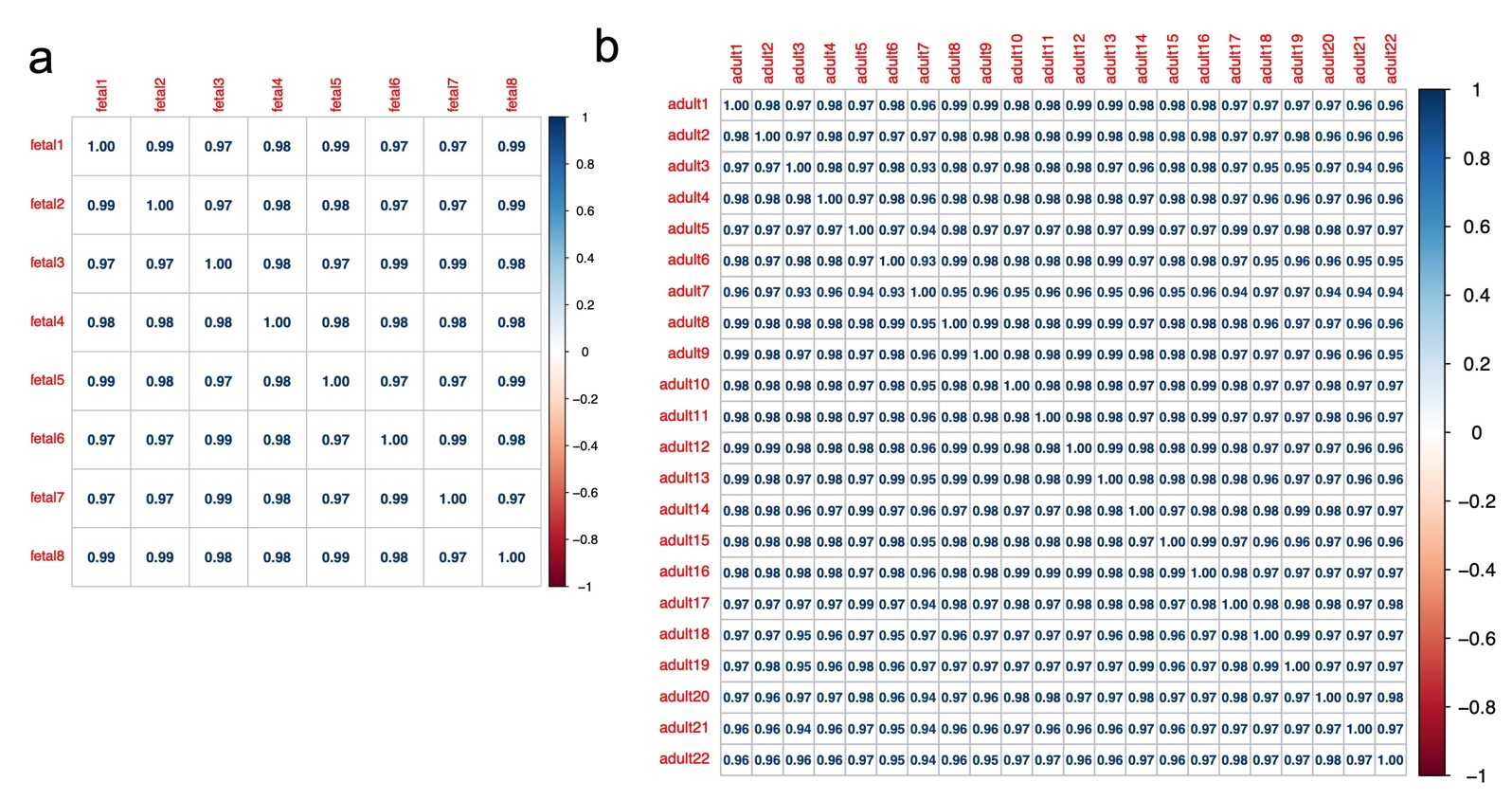
Supplementary Figures**

**Figure S1.** Pearson correlation plots for the DNA methylation profiling of non-cultured human **a.** fetal (n=8) and **b.** adult chondrocytes (n=22).

**
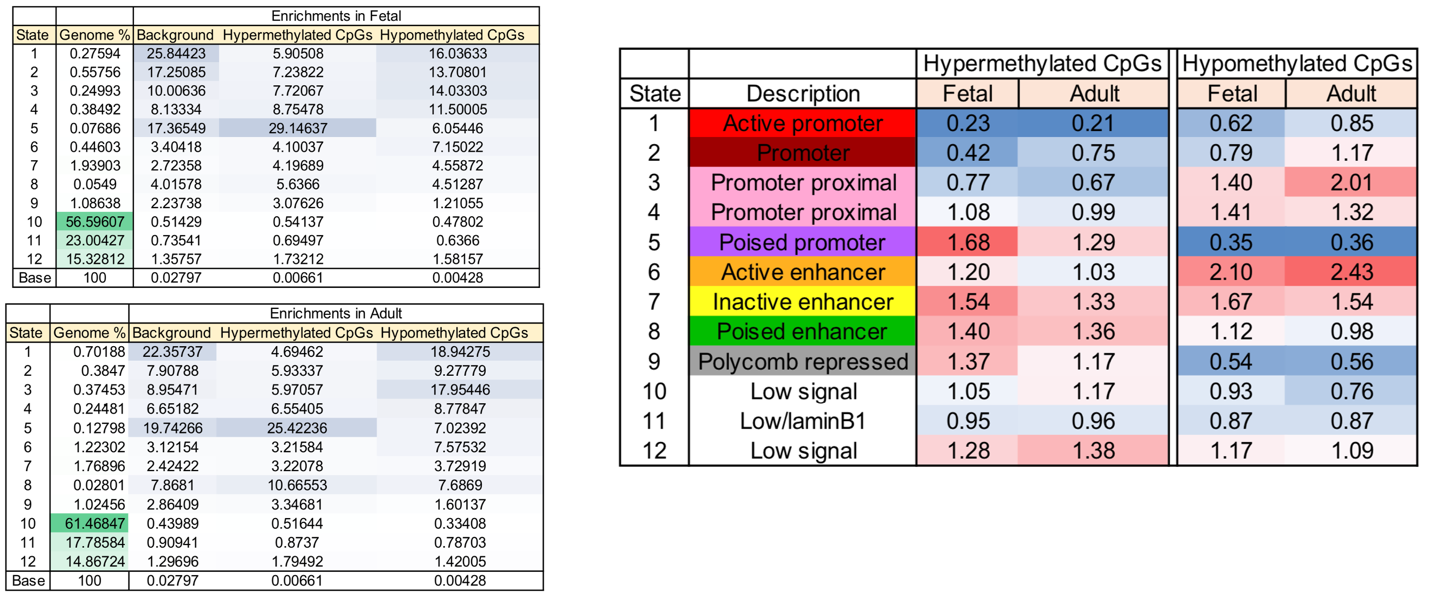
**

**Figure S2.** Age-correlated CpGs are associated with distinct chromatin states. ChromHMM model shows enrichment of the 12 chromatin states for age-correlated CpGs in fetal and adult chondrocytes. Hypomethylated CpGs refer to CpG sites which are losing methylation with chondrocyte age. Hypermethylated CpGs refer to CpG sites which are gaining methylation with chondrocyte age. Emission probabilities (left panel) shows the occurrence of CpGs in each chromatin state. Rows correspond to chromatin states. The occurrence of CpGs in each chromatin state is represented by color code: 0(white) to 100(blue). Chromatin state enrichments (right panel) shows the enrichment score for CpGs in each chromatin state. A 3-color code was used to represent the range of enrichment score: Lowest value(blue), 50percentile(white) and Highest value(red).

**
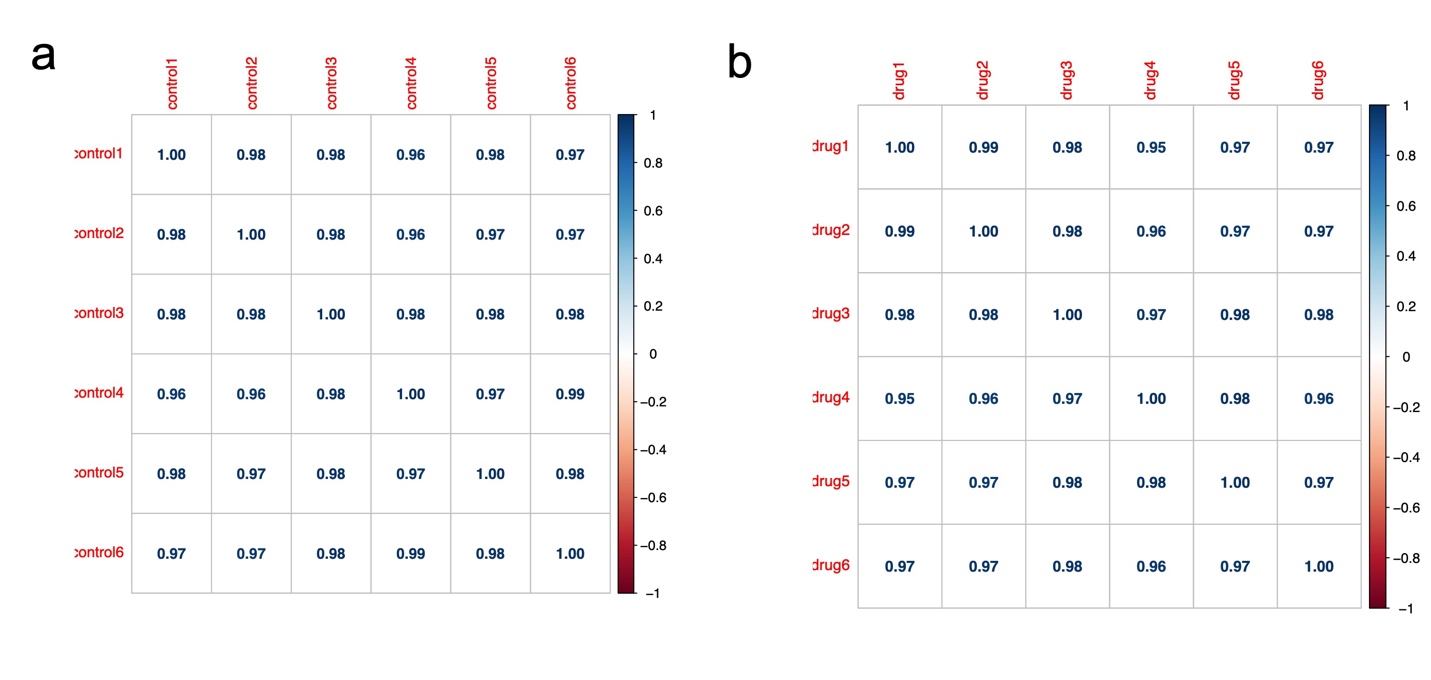
**

**Figure S3.** Pearson correlation plots for the DNA methylation profiling of **a.** control (n=6) and **b.** 423F treated (n=6) aged human adult chondrocytes.

**Figure S4.** qRT-PCR data analysis for STAT3 in scrambled and STAT3 shRNA fetal chondrocytes. Statistical analysis was performed using 2-tailed Student’s t test in GraphPad Prism 9.0 and p-value <0.05 was considered as statistically significant. Mean with standard deviation is plotted.


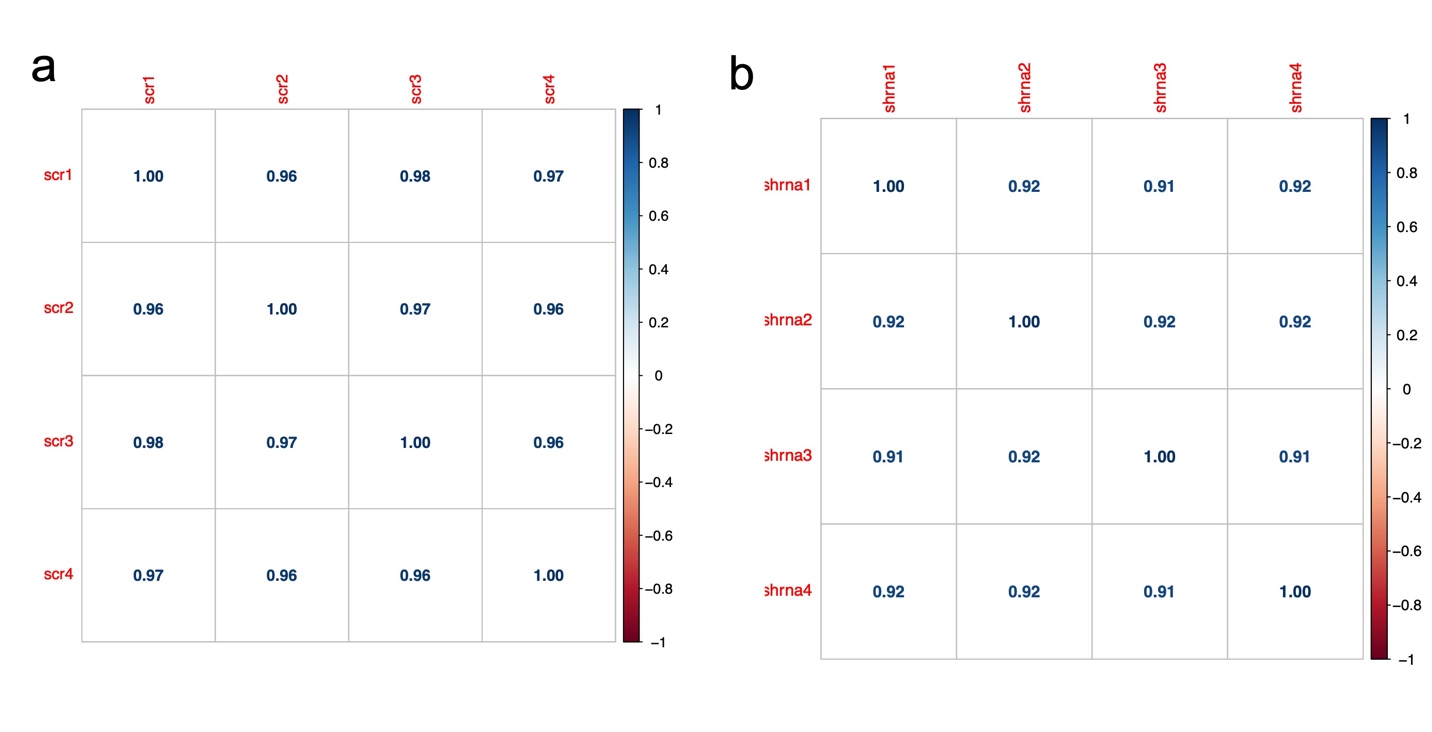


**Figure S5.** Pearson correlation plots for the DNA methylation profiling of **a.** scrambled control (n=4) and **b.** STAT3 shRNA (n=4) transduced fetal chondrocytes.

**Figure S6.** Active STAT3 is highly expressed in osteoarthritic chondrocytes as compared to healthy adult chondrocytes. A representative Western Blot (N=6 biological replicates).

**
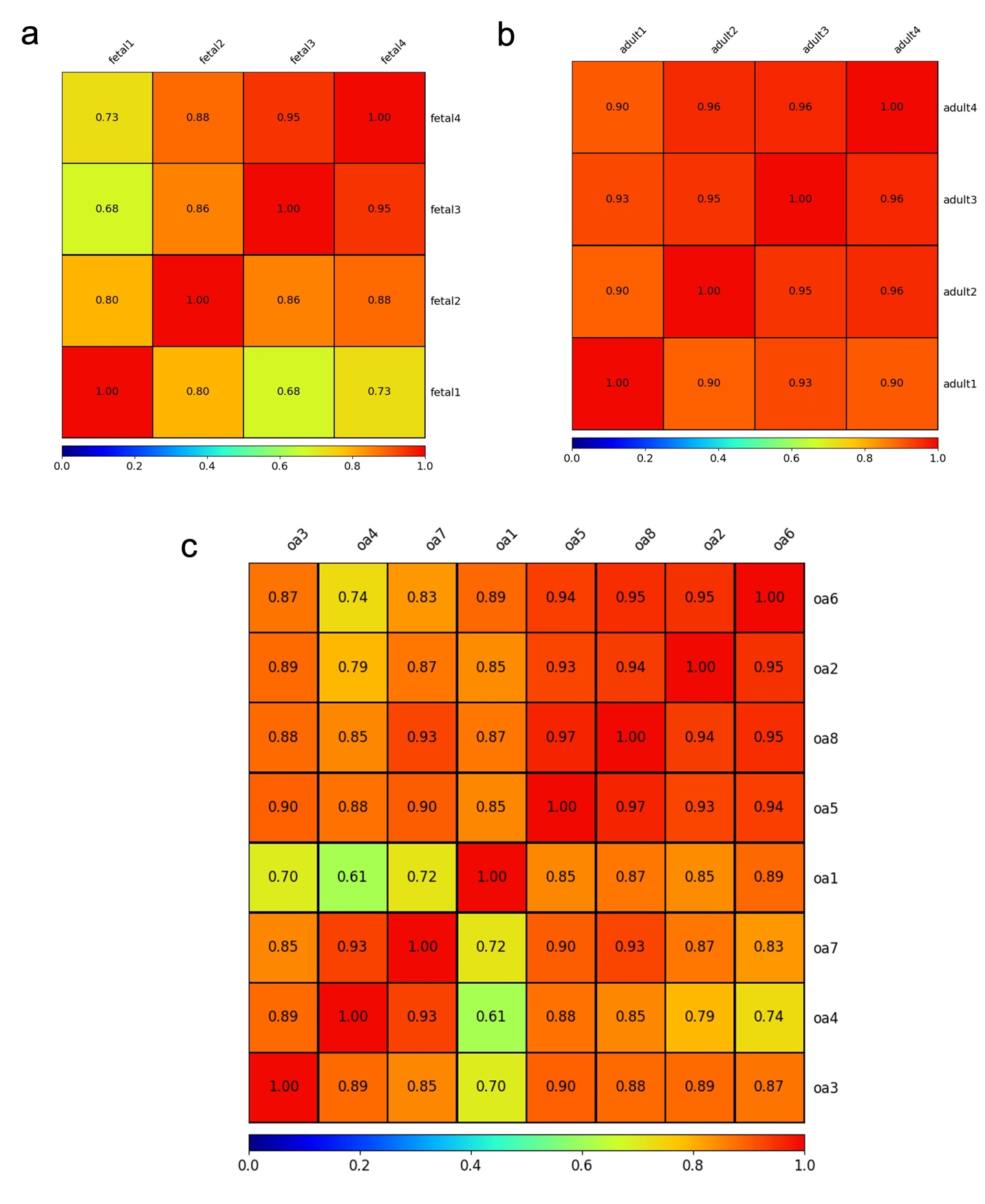
Figure S7.** Pearson correlation plots for CUT&RUN assays of **a.** fetal (n=4) **b.** healthy adult (n=4) and **c.** osteoarthritic (n=8) chondrocytes.


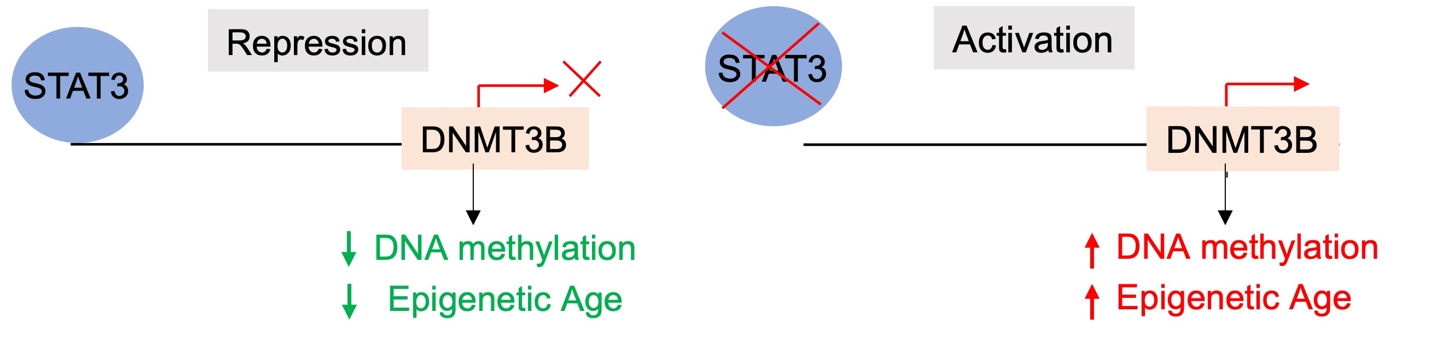


**Figure S8.** A model showing the plausible regulation of DNA methylation by STAT3 via DNMT3B.
